# Cardiolipin Alters *Rhodobacter sphaeroides* Cell Shape by Affecting Peptidoglycan Precursor Biosynthesis

**DOI:** 10.1128/mBio.02401-18

**Published:** 2019-02-19

**Authors:** Ti-Yu Lin, William S. Gross, George K. Auer, Douglas B. Weibel

**Affiliations:** a Department of Biochemistry, University of Wisconsin−Madison, Madison, Wisconsin, USA; b Department of Biomedical Engineering, University of Wisconsin−Madison, Madison, Wisconsin, USA; c Department of Chemistry, University of Wisconsin−Madison, Madison, Wisconsin, USA; University of Hawaii at Manoa

**Keywords:** MreB, MurG, cardiolipin, cell shape, peptidoglycan

## Abstract

Cardiolipin (CL) is an anionic phospholipid that plays an important role in regulating protein biochemistry in bacteria and mitochondria. Deleting the CL synthase gene (Δ*cls*) in Rhodobacter sphaeroides depletes CL and decreases cell length by 20%. Using a chemical biology approach, we found that a CL deficiency does not impair the function of the cell wall elongasome in R. sphaeroides; instead, biosynthesis of the peptidoglycan (PG) precursor lipid II is decreased. Treating R. sphaeroides cells with fosfomycin and d-cycloserine inhibits lipid II biosynthesis and creates phenotypes in cell shape, PG composition, and spatial PG assembly that are strikingly similar to those seen with R. sphaeroides Δ*cls* cells, suggesting that CL deficiency alters the elongation of R. sphaeroides cells by reducing lipid II biosynthesis. We found that MurG—a glycosyltransferase that performs the last step of lipid II biosynthesis—interacts with anionic phospholipids in native (i.e., R. sphaeroides) and artificial membranes. Lipid II production decreases 25% in R. sphaeroides Δ*cls* cells compared to wild-type cells, and overexpression of MurG in R. sphaeroides Δ*cls* cells restores their rod shape, indicating that CL deficiency decreases MurG activity and alters cell shape. The R. sphaeroides Δ*cls* mutant is more sensitive than the wild-type strain to antibiotics targeting PG synthesis, including fosfomycin, d-cycloserine, S-(3,4-dichlorobenzyl)isothiourea (A22), mecillinam, and ampicillin, suggesting that CL biosynthesis may be a potential target for combination chemotherapies that block the bacterial cell wall.

## Abstract (precis)

The phospholipid composition of the cell membrane influences the spatial and temporal biochemistry of cells. We studied molecular mechanisms connecting membrane composition to cell morphology in the model bacterium Rhodobacter sphaeroides. The peptidoglycan (PG) layer of the cell wall is a dominant component of cell mechanical properties; consequently, it has been an important antibiotic target. We found that the anionic phospholipid cardiolipin (CL) plays a role in determination of the shape of R. sphaeroides cells by affecting PG precursor biosynthesis. Removing CL in R. sphaeroides alters cell morphology and increases its sensitivity to antibiotics targeting proteins synthesizing PG. These studies provide a connection to spatial biochemical control in mitochondria, which contain an inner membrane with topological features in common with R. sphaeroides.

## INTRODUCTION

**FIG 1:**
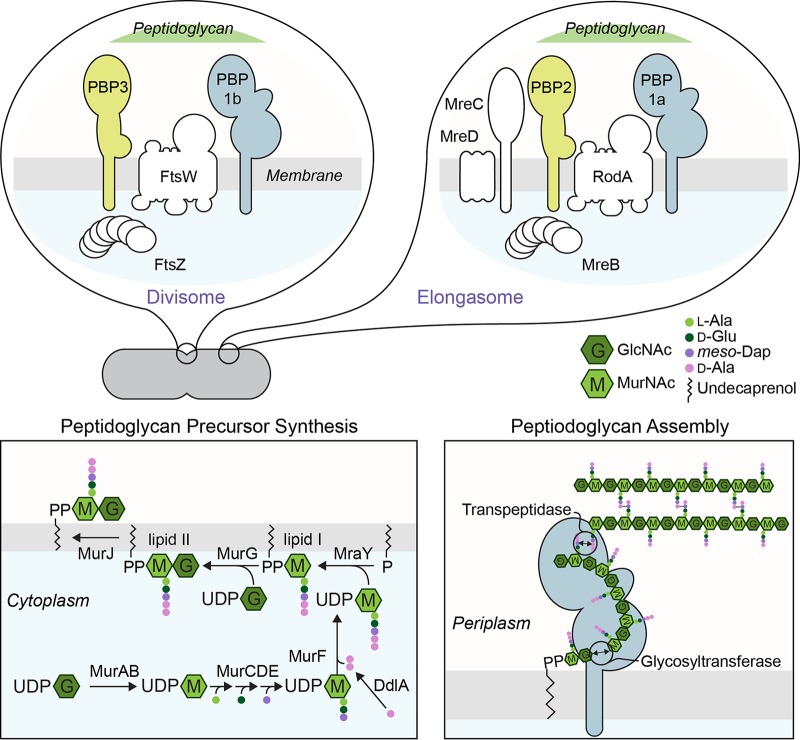
PG synthesis in Gram-negative bacteria. PG synthesis in Gram-negative bacteria is conserved and proceeds in three distinct steps. The process starts with the biosynthesis of monosaccharide-peptide precursors in the cytoplasm followed by the formation of lipid I and lipid II at the cell membrane. Lipid II is then oriented from the cytoplasm to the periplasm, where PG assembly occurs. In rod-shaped bacteria, cytoskeletal proteins guide the location of PG synthesis at different stages of the cell cycle. During cell division, FtsZ organizes the divisome, which synthesizes PG at the division site. For clarity, we include only a subset of cell division proteins in the diagram. During cell elongation, the elongasome is associated with MreB, which directs PG synthesis at the sidewalls. See text for details. GlcNAc, *N*-acetylglucosamine; MurNAc, *N*-acetylmuramic acid; Ala, alanine; Glu, glutamic acid; Dap, *meso*-diaminopimelic acid.

Bacterial cells have diverse shapes that play roles in their adaptation and survival, including motility, adhesion, replication, pathogenicity, and evasion of the mammalian immune system (1, 2). The shape of bacteria arises from the morphology of the cell wall and specifically from the layer of peptidoglycan (PG) surrounding the cytoplasmic membrane that resists osmotic pressure and other mechanical forces applied to cells (3, 4). Researchers in the field of antibiotic development have paid acute attention to the underlying machinery controlling PG assembly and the catastrophic effect of inhibiting its biosynthesis (5, 6). PG is synthesized in three distinct steps in Gram-negative bacteria (Fig. 1). (i) Monosaccharide-peptide precursors are synthesized in the cytoplasm by seven enzymes: MurABCDEF and DdlA. (ii) A lipid attached by MraY tethers this precursor to the inner leaflet of the cytoplasmic membrane (referred to as lipid I), a second sugar is attached by MurG to create a disaccharide-peptide precursor (referred to as lipid II), and the molecule is flipped to the outer leaflet of the bilayer by MurJ. (iii) Lipid II is polymerized in the periplasmic space by a glycosyltransferase (GTase) and the nascent glycan strands are incorporated into the existing PG by a transpeptidase (TPase) that cross-links peptides on adjacent lipid II molecules.

In rod-shaped bacteria, PG synthesis occurs at different sites in the cell at different stages of the cell cycle. During cell division, FtsZ initiates the organization of the divisome containing PBP1b (a bifunctional GTase and TPase) and PBP3 (a TPase that requires FtsW for its function), which perform PG assembly at the division site. During cell elongation, MreB forms a scaffold for assembly of an elongasome containing PBP1a (a bifunctional GTase and TPase) and PBP2 (a TPase that requires RodA for its activity), which catalyze PG assembly along the sidewalls of the cell (3, 4).

Previous studies demonstrating a connection between membrane composition and bacterial biochemistry (7) motivated us to explore the role of the phospholipid bilayer in synthesis of PG and determination of bacterial cell shape. We previously observed that a mutant (strain Δ*cls*) of the Gram-negative, rod-shaped bacterium Rhodobacter sphaeroides lacking the anionic, intrinsically curved phospholipid cardiolipin (CL) creates cells that are shorter in length than those created by the wild-type (wt) strain (8). A similar change in cell morphology has not been observed in other rod-shaped bacterial cells lacking CL, e.g., Escherichia coli and Bacillus subtilis (9, 10), suggesting that this phenotype may be unique to alphaproteobacteria. In this paper, we describe R. sphaeroides using a strategy similar to that used by other bacteria—i.e., MreB-directed cell elongation—to determine the rod shape of cells. Despite a reduction in growth along its cylindrical cell body, the CL-deficient mutant of R. sphaeroides has an elongasome that functions normally. We found that the influence of the depletion of CL on R. sphaeroides cell shape arises from effects on the biosynthesis of the PG precursor, lipid II. We describe experiments supporting the hypothesis that a CL deficiency in R. sphaeroides reduces the supply of lipid II to the elongasome for PG assembly at the sidewalls of the cell, which alters cell shape and makes cells susceptible to PG-targeting antibiotics.

## RESULTS

### CL plays a role in determining R. sphaeroides cell shape.

**FIG 2:**
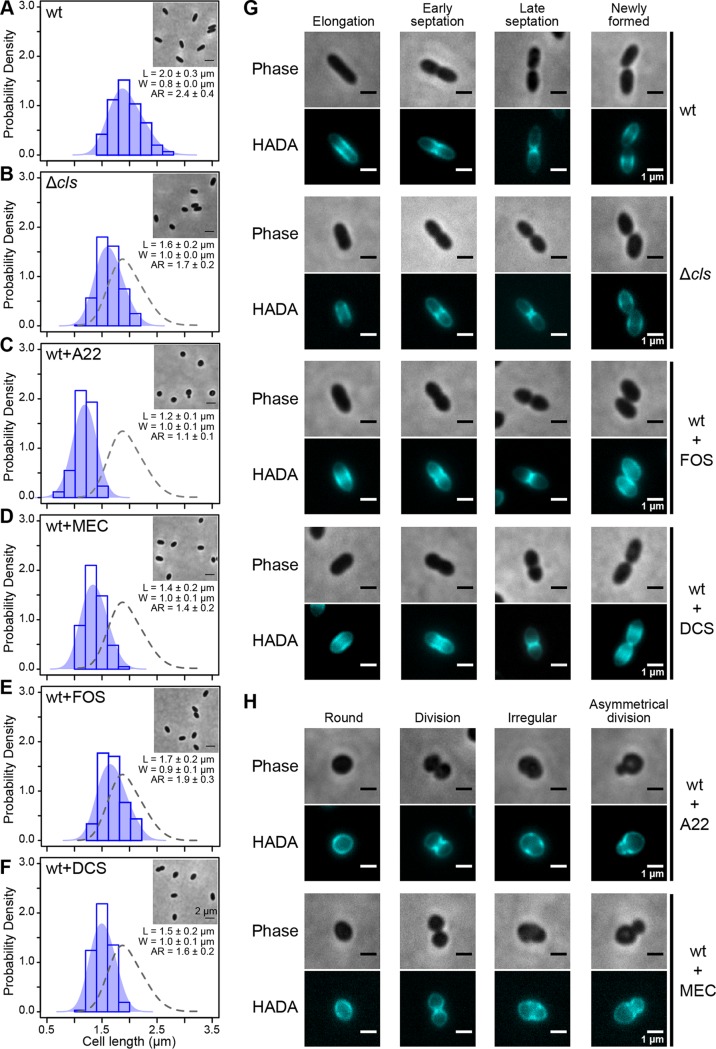
CL participates in cell shape determination by affecting lipid II biosynthesis. (A to F) Probability density histogram of the cell length distribution of R. sphaeroides wt cells (A), Δ*cls* cells (B), wt cells treated with A22 (C), wt cells treated with mecillinam (MEC) (D), wt cells treated with fosfomycin (FOS) (E), and wt cells treated with d-cycloserine (DCS) (F). Cells were grown in plain Sistrom’s minimal medium or Sistrom’s succinate medium containing the indicated small-molecule inhibitors until log phase (absorbance of 0.6, λ = 600 nm) and imaged by phase-contrast bright-field microscopy. Scale bar, 2 μm. Each data point represents a mean value ± standard deviation of the cell length (L), width (W), and aspect ratio (AR) for 300 cells determined by ImageJ. The shaded blue area overlaying the histogram represents the Kernel density estimation (KDE) of the cell length distribution. We overlaid a gray dashed line outlining the KDE of the cell length distribution of R. sphaeroides wt cells with the other five histograms for comparison. (G) Representative micrographs of HADA-labeled R. sphaeroides wt cells, Δ*cls* cells, wt cells treated with FOS, and wt cells treated with DCS. (H) Representative micrographs of HADA-labeled R. sphaeroides wt cells treated with A22 and wt cells treated with MEC. Cells were grown in plain medium or medium containing the indicated small-molecule inhibitors to early log phase (absorbance of 0.3, λ = 600 nm), labeled with HADA, and imaged using phase-contrast bright-field and fluorescence microscopy. We used sub-MICs of the following inhibitors: A22 (10 μg/ml), MEC (0.5 μg/ml), FOS (250 μg/ml), and DCS (0.05 μg/ml).

The R. sphaeroides cell membrane contains four major classes of phospholipids, namely, phosphatidylethanolamine (PE), phosphatidylcholine (PC), phosphatidylglycerol (PGL), and CL, in a ratio of approximately 5:2:2.4:0.6 (PE/PC/PGL/CL) by weight (11). A CL-deficient mutant of R. sphaeroides lacking the *CL* synthase gene (strain Δ*cls*) produces a reduced amount of CL (0.5% of total lipids, representing an ∼90% decrease in CL) and displays a cell shape phenotype with a characteristic decrease (20%) in cell length compared to the wt strain (Fig. 2A and B) (8, 11). To investigate whether the change in the cell shape of the R. sphaeroides Δ*cls* mutant is due to an increased cell division rate (relative to cell growth), we performed single-cell time-lapse imaging of both R. sphaeroides wild-type (wt) and Δ*cls* cells (Fig. S1 in the supplemental material). We imaged cells, tracked their growth directly between cell division events, and found that the wt and Δ*cls* strains had comparable doubling times of ∼150 min. The Δ*cls* mutant, however, displayed a 40% decrease in the rate of cell elongation compared to the wt strain, suggesting that the morphological change in the R. sphaeroides Δ*cls* mutant may arise from a defect in cell elongation. Complementation of the Δ*cls* mutant by ectopically expressing CL synthase in cells from the IPTG (isopropyl-β-d-thiogalactopyranoside)-inducible expression vector pIND (12) increased the concentration of CL to 2.3% of total lipids (∼50% of the level in wt cells) and restored the cell elongation rate by 20% (Fig. S1), suggesting that CL contributes to sculpting the rod shape of R. sphaeroides cells.

10.1128/mBio.02401-18.1 FIG S1 R. sphaeroides Δ*cls* cells have a lower cell elongation rate than wt cells. (A to C) Probability density histograms of the cell length distribution of R. sphaeroides wt cells (A), Δ*cls* cells (B), and Δ*cls* cells expressing Cls (C). Cells in log phase (absorbance of 0.6, λ = 600 nm) were imaged by phase-contrast bright-field microscopy. Scale bar, 2 μm. Each data point represents a mean value ± standard deviation of the cell length (L), width (W), and aspect ratio (AR) for 300 cells determined by ImageJ. The shaded blue area overlaying the histogram represents the Kernel density estimation (KDE) of the cell length distribution. We overlaid a gray dashed line outlining the KDE of the cell length distribution of R. sphaeroides wt cells with the other two histograms for comparison. Levels of CL in cells were determined from a thin-layer chromatography (TLC) plate. Each data point (mean value ± standard deviation) was obtained from three independent experiments. For details, see references 8 and 11. (D) Representative time-lapse micrographs of R. sphaeroides wt, Δ*cls*, and Δ*cls* cells expressing Cls. Cells in early log phase (absorbance of 0.3, λ = 600 nm) were imaged using phase-contrast bright-field microscopy. We determined the cell elongation rate (Δ*L*/Δ*t*) by the formula (*L*_90_ – *L*_0_)/1.5 (μm/h), where *L*_90_ is the cell length at 90 min and *L*_0_ is the cell length at 0 min. Each data point (mean value ± standard deviation) was determined by imaging ≥23 cells and using ImageJ to determine cell length. Download FIG S1, TIF file, 0.6 MB. Copyright © 2019 Lin et al. 2019 Lin et al. This content is distributed under the terms of the Creative Commons Attribution 4.0 International license.

### CL deficiency does not impair R. sphaeroides elongasome function.

MreB is a bacterial cytoskeletal protein that is homologous to eukaryotic actin (13). In rod-shaped bacteria, MreB polymerizes into filamentous structures positioned on the inner leaflet of the cytoplasmic membrane that is associated with elongasome components, including the cell elongation-specific TPase PBP2. MreB filaments move circumferentially around the cell with a velocity that is correlated to the rate of PG assembly and in an orientation that may guide elongation of the PG layer of the cell wall (14–16). In R. sphaeroides, MreB, MreC, MreD, PBP2, and RodA are hypothetically encoded at the same locus (Fig. S2A) (17). MreB requires MreC and MreD for its cellular localization, and PBP2 requires RodA for its TPase activity (3). RodA has recently been reported to also possess GTase activity (18, 19). We used a chemical biology approach to investigate whether R. sphaeroides uses a elongasome strategy for shape-determination similar to that summarized above. We treated R. sphaeroides cells with the small-molecule inhibitors S-(3,4-dichlorobenzyl)isothiourea (A22; binds MreB and disassembles MreB filaments) and mecillinam (MEC; binds PBP2 and inhibits its TPase activity required for cell elongation). The MIC of A22 was 60 μg/ml for R. sphaeroides, and the MIC of MEC was 3 μg/ml. Treatment using sub-MICs of both drugs (A22, 10 μg/ml; MEC, 0.5 μg/ml) did not significantly impair R. sphaeroides cell growth and yet decreased cell length (A22, 40%; MEC, 30%), which matches the phenotype of these compounds in other rod-shaped bacteria (3) and suggests a conserved cell elongation machinery in R. sphaeroides (Fig. 2C and D). We observed a similar phenotype (i.e., shorter cell length) in comparisons between R. sphaeroides cells with reduced CL concentrations and cells treated with A22 and MEC; this result led us to hypothesize that an altered elongasome function in the R. sphaeroides Δ*cls* mutant may reduce PG assembly at the sidewalls of the cell.

10.1128/mBio.02401-18.2 FIG S2 CL deficiency does not affect the expression level of the elongasome in R. sphaeroides. (A) Schematic diagram showing *mre* and *mrd* loci in the R. sphaeroides genome. The *mre* locus is composed of *mreB*, *mreC*, and *mreD*; the *mrd* locus contains *pbp2* and *rodA*. These two loci cluster together and are hypothesized to be organized as a single operon in the R. sphaeroides genome. (B) The expression levels of elongasome genes in R. sphaeroides wt and Δ*cls* cells were assayed by qPCR. Shown are mean values ± standard deviations obtained from three independent experiments, each performed in triplicate. All the differences (<50%) are considered to be insignificant. Download FIG S2, TIF file, 0.05 MB. Copyright © 2019 Lin et al. 2019 Lin et al. This content is distributed under the terms of the Creative Commons Attribution 4.0 International license.

We used real-time quantitative PCR (qPCR) to test if transcription of genes coding for elongasome components is altered in R. sphaeroides Δ*cls* cells. We found no significant difference in the levels of transcription of elongasome genes in wt and Δ*cls* cells (Fig. S2B). We also treated R. sphaeroides cells with the PBP1a inhibitor cefsulodin to inhibit its TPase activity. The MIC of cefsulodin for R. sphaeroides cells is 20 μg/ml; a sub-MIC (5 μg/ml) of the drug did not cause significant growth defects or changes in cell shape (Fig. S3), suggesting that PBP1a does not participate in cell elongation (19).

10.1128/mBio.02401-18.3 FIG S3 Inhibition of PBP1a does not cause a change in R. sphaeroides cell shape. A probability density histogram of the cell length distribution of R. sphaeroides wt cells treated with cefsulodin is shown. Cells were grown in medium containing 5 μg/ml cefsulodin until they reached log phase (absorbance of 0.6, λ = 600 nm) and imaged by phase-contrast bright-field microscopy. Scale bar, 2 μm. Each data point represents a mean value ± standard deviation of the cell length (L), width (W), and aspect ratio (AR) for 300 cells determined by ImageJ. The shaded blue area overlaying the histogram represents the Kernel density estimation (KDE) of the cell length distribution. We overlaid a gray dashed line outlining the KDE of the cell length distribution of R. sphaeroides wt cells with the histogram for comparison. Download FIG S3, TIF file, 0.1 MB. Copyright © 2019 Lin et al. 2019 Lin et al. This content is distributed under the terms of the Creative Commons Attribution 4.0 International license.

MreB filaments are involved in the process of PG assembly at the sidewalls of cells during cell elongation. We examined whether the structure and function of MreB are disrupted in R. sphaeroides Δ*cls* cells by comparing the PG growth patterns of R. sphaeroides wt and Δ*cls* strains. We probed PG assembly in cells at different stages of the cell cycle with HADA, a fluorescent analog of d-alanine containing a coumarin moiety that can be covalently incorporated into newly synthesized PG (20, 21). PG assembly in R. sphaeroides wt cells occurred along the sidewalls and accompanied cell elongation (Fig. 2G). At early and late stages of cell division (i.e., cell septation), PG assembly is focused at the division plane. After the mother and daughter cells are separated, PG assembly returns to the sidewalls as cells elongate. This cycle of PG assembly was not observed in R. sphaeroides wt cells treated with A22 in which the structure of MreB was disrupted (Fig. 2H); A22-treated R. sphaeroides cells exhibited three aberrant phenotypes: round shapes, irregular shapes, and asymmetric cell division. Round R. sphaeroides cells displayed peripheral PG assembly and divided at the mid-cell. PG was assembled at apparently random positions in cells with irregular shapes. Cells undergoing asymmetrical division displayed asymmetrical labeling of septal PG. R. sphaeroides wt cells treated with MEC had phenotypes similar to those of A22-treated cells, suggesting that inhibiting PBP2 activity may disrupt the structure of MreB or retard the movement of MreB filaments (Fig. 2H). The pattern of PG assembly in R. sphaeroides Δ*cls* cells was similar to that in wt cells (Fig. 2G), suggesting that a change in shape is unlikely to arise from disrupting the MreB structure or inhibiting PBP2 activity.

### Inhibition of lipid II biosynthesis results in a phenotype that resembles that of R. sphaeroides Δ*cls* cells.

In rod-shaped bacteria, lipid II biosynthesis colocalizes with PG assembly at different stages of the cell cycle (22). The change in cell shape that we observed in the R. sphaeroides Δ*cls* mutant may have arisen from a reduction in lipid II biosynthesis. To test this hypothesis, we reduced lipid II biosynthesis by treating R. sphaeroides cells with the MurA inhibitor fosfomycin (FOS) and the DdlA inhibitor d-cycloserine (DCS). MurA converts UDP-*N*-acetylglucosamine (UDP-GlcNAc) into UDP-N-acetylmuramic acid (UDP-MurNAc) in the first concerted step in lipid II biosynthesis. DdlA is a d-alanine (d-Ala) ligase that produces d-Ala-d-Ala for incorporation into lipid I (Fig. 1). The MICs of FOS and DCS for R. sphaeroides were 1.5 mg/ml and 1 μg/ml, respectively. R. sphaeroides cells treated with sub-MICs of either drug (FOS, 250 μg/ml; DCS, 0.05 μg/ml) had a decrease in cell length (FOS, 15%; DCS, 25%) and lacked significant growth defects (Fig. 2E and F). HADA labeling experiments indicated that these two drugs did not affect the pattern of PG assembly in R. sphaeroides cells (Fig. 2G), suggesting that a change in the shape of R. sphaeroides Δ*cls* cells may arise from a decrease in lipid II biosynthesis arising from a CL deficiency.

**FIG 3:**
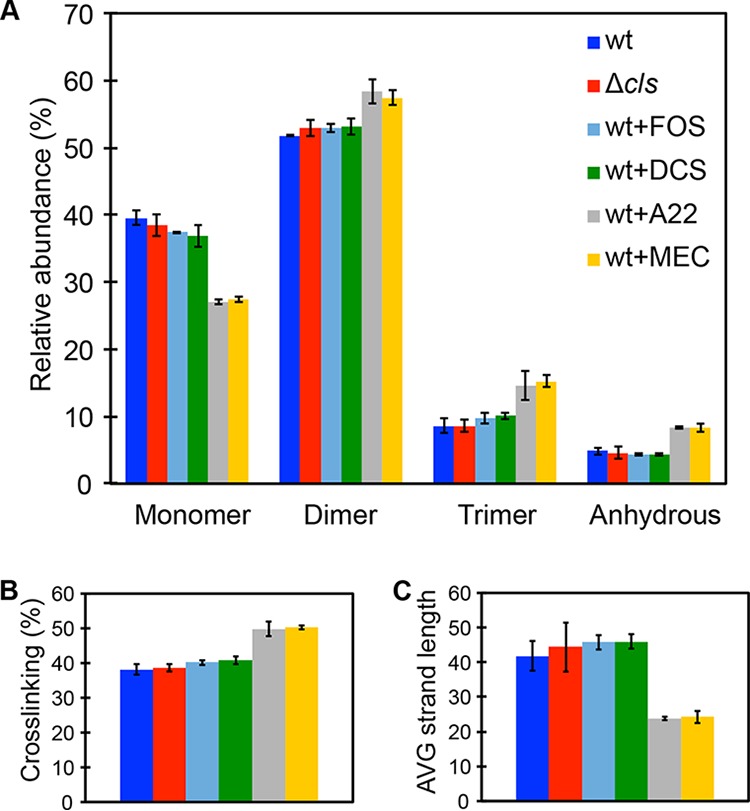
CL deficiency does not affect the PG composition of cells. (A) Quantification of muropeptides purified from R. sphaeroides wt cells, Δ*cls* cells, wt cells treated with A22, wt cells treated with MEC, wt cells treated with FOS, and wt cells treated with DCS. A22 and MEC treatment decreased the relative abundance of muropeptide monomer by 33%, increased the relative abundance of crossed-linked muropeptides (dimer and trimer) by 20%, and increased the relative abundance of anhydrous muropeptides by 60%; all values are compared to wt cells. (B and C) These alterations in muropeptide composition yielded a PG with a higher level of cross-linking (B) and a shorter average strand length (C) than were seen with the wt cells. CL deficiency did not change the PG composition of R. sphaeroides Δ*cls* cells, which resembled those of wt cells and wt cells treated with either FOS or DCS. Data represent mean values ± standard deviations obtained from three independent experiments. We used sub-MICs of the following inhibitors: A22 (10 μg/ml), MEC (0.5 μg/ml), FOS (250 μg/ml), and DCS (0.05 μg/ml).

To test this hypothesis, we determined the PG composition of R. sphaeroides cells using ultraperformance liquid chromatography-mass spectrometry (UPLC-MS) (Table 1; see also Fig. S4). We treated R. sphaeroides wt cells with A22 and MEC, isolated PG, digested it with mutanolysin (an N-acetylmuramidase), analyzed the material by UPLC-MS (23), and found that both compounds changed the PG composition of cells (Fig. 3A). Compared to wt cells, treatment with either A22 or MEC decreased the concentration of disaccharide-peptide (muropeptide) monomer by 33% and increased the concentration of crossed-linked muropeptides (dimer and trimer) by 20%, resulting in a 32% increase in the degree of cross-linking (Fig. 3B). Treatment with both A22 and MEC led to a 60% increase in the concentration of anhydromuramyl muropeptides present at the terminating end of the glycan strand, reflecting a 43% decrease in the average glycan strand length (Fig. 3C) (24). Similar results were found in E. coli cells treated with A22 (25). Surprisingly, R. sphaeroides Δ*cls* cells have a PG composition indistinguishable from that seen in wt cells or in wt cells treated with FOS or DCS (Fig. 3), suggesting that CL deficiency does not impair the elongasome function and that a change in cell shape of the R. sphaeroides Δ*cls* mutant may arise from a reduction in lipid II biosynthesis.

**TABLE 1:** Muropeptides analyzed by UPLC-MS in positive-ion mode

Peak^*a*^	Retention time (min)	Calculated mass	Observed *m*/*z*	Length of the stem peptides
1	7.6	870.37	871.37 (+1)	Tri
2	9	698.29	699.29 (+1)	Di
3	12	941.41	942.41 (+1)	Tetra
4a	13.7	1,722.71	575.23 (+3)	Tri-tri
4b	20.3	1,722.71	862.35 (+2)	Tri-tri
5a	21.7	1,793.77	897.88 (+2)	Tri-tetra
5b	22.8	1,793.77	897.88 (+2)	Tri-tetra
6	24.1	1,864.81	933.40 (+2)	Tetra-tetra
7a	28	2,717.15	906.71 (+3)	Tri-tetra-tetra
7b	29.2	2,717.15	906.71 (+3)	Tri-tetra-tetra
8	30.1	2,788.21	930.40 (+3)	Tetra-tetra-tetra
9a	30.7	1,773.74	887.87 (+2)	Anhydro tri-tetra
9b	31.9	1,773.74	887.87 (+2)	Anhydro tri-tetra
10	33.2	1,844.77	923.38 (+2)	Anhydro tetra-tetra

a Refer to chromatograms in Fig. S4.

10.1128/mBio.02401-18.4 FIG S4 Chromatograms of purified muropeptides from R. sphaeroides wt cells, Δ*cls* cells, wt cells treated with FOS, wt cells treated with DCS, wt cells treated with A22, and wt cells treated with MEC. Cell walls were digested, purified, and analyzed by UPLC-MS. Identified peaks are provided in Table 1. Quantification of peaks is shown in Fig. 3. The asterisk (*) denotes a peak of undesirable contaminants in the column. Download FIG S4, TIF file, 0.2 MB. Copyright © 2019 Lin et al. 2019 Lin et al. This content is distributed under the terms of the Creative Commons Attribution 4.0 International license.

### CL affects lipid II biosynthesis.

**FIG 4:**
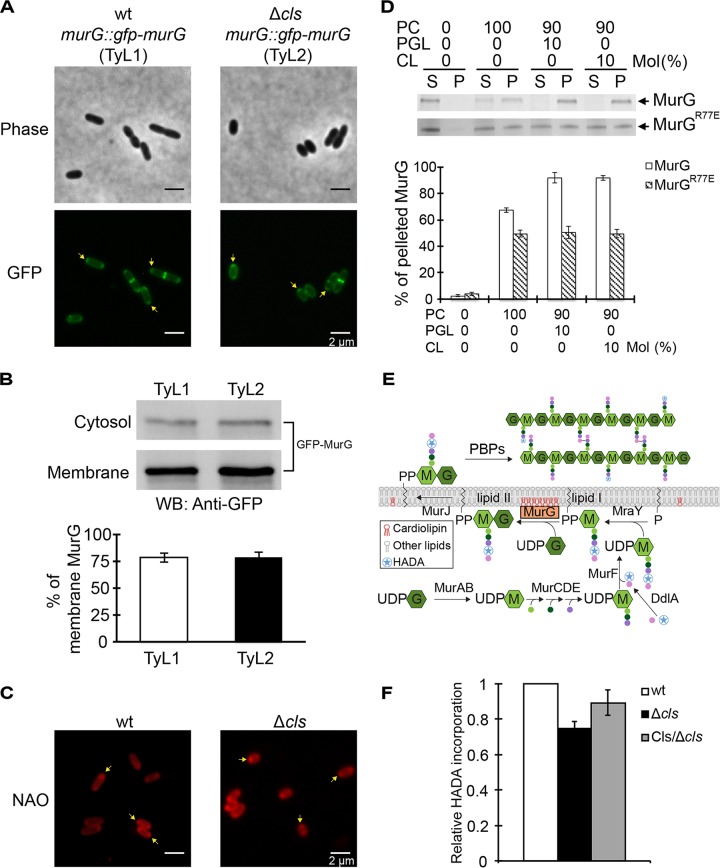
R. sphaeroides MurG interacts with CL, and the interaction is important for its enzymatic activity and cell elongation. (A) Representative micrographs of TyL1 and TyL2 cells, which are R. sphaeroides wt and Δ*cls* cells in which the genomic *murG* was replaced with *gfp-murG*, respectively. Cells were grown to log phase (absorbance of 0.6, λ = 600 nm) and imaged using phase-contrast bright-field and fluorescence microscopy. Arrows indicate the pole-localized MurG. (B) Biochemical fractionation of TyL1 and TyL2 cells. We fractionated TyL1 and TyL2 cell lysates that had the same amount of proteins into cytosolic and membrane fractions and performed Western blot (WB) analysis using a monoclonal antibody against GFP. The percentage of membrane-localized MurG was determined by quantifying the optical densitometry signal using ImageJ. Data represent mean values ± standard deviations obtained from three independent experiments. (C) Representative micrographs of R. sphaeroides wt and Δ*cls* cells stained with NAO. Cells were grown to early log phase (absorbance of 0.3, λ = 600 nm), stained with NAO, and imaged using fluorescence microscopy. Arrows indicate the polar enrichment of NAO. (D) Liposome-pelleting assays of MurG and MurG^R77E^ with liposomes containing the indicated phospholipids. The percentage of pelleted MurG was determined by quantifying the optical densitometry signal using ImageJ. Data represent mean values ± standard deviations obtained from three independent experiments. S, supernatant; P, pellet. (E) A diagram depicting the incorporation of HADA into the lipid II molecule by MurG and its assembly into the existing PG by PBP proteins. We hypothesize that CL interacts with MurG and is important for its activity. As CL does not affect PG assembly, we assessed the effect of CL on lipid II production by quantifying the incorporation of HADA into the existing PG. (F) Quantification of HADA incorporation in R. sphaeroides wt cells, Δ*cls* cells, and Δ*cls* cells expressing Cls. Cells were grown until early log phase (absorbance of 0.3, λ = 600 nm) and labeled with HADA. We measured the fluorescence emission of HADA and normalized the fluorescent signals by determination of CFU. Each data point (mean value ± standard deviation) was obtained from three independent experiments.

We found no significant differences in the levels of transcription of genes coding for enzymes that synthesize lipid II in R. sphaeroides wt and Δ*cls* cells (Fig. S5). We then investigated the impact of CL on the activity of specific enzymes involved in lipid II biosynthesis. MurG is a GTase and a hypothetical peripheral membrane protein that catalyzes the rate-limiting step of lipid II formation by transferring the GlcNAc from UDP-GlcNAc to lipid I (Fig. 1) (26, 27). We fused *gfp* to the genomic *murG* in both R. sphaeroides wt and Δ*cls* strains (resulting in TyL1 and TyL2 strains, respectively; Fig. S6) and imaged the cells. During elongation, MurG localized to the membrane and concentrated at the cell poles. During division, MurG localized at the division plane (Fig. 4A). This subcellular MurG localization pattern was unchanged in R. sphaeroides Δ*cls* cells. Cell fractionation experiments indicated no significant difference between the amounts of membrane-localized MurG (∼75%) in R. sphaeroides wt cells and Δ*cls* cells (Fig. 4B). Apparently, membrane localization of MurG does not require CL and changes in R. sphaeroides Δ*cls* cell shape are not due to MurG mislocalization.

10.1128/mBio.02401-18.5 FIG S5 CL deficiency does not affect expression levels of the genes coding for enzymes responsible for PG precursor biosynthesis in R. sphaeroides. The expression levels of lipid II synthase genes in R. sphaeroides wt and Δ*cls* cells were assayed by qPCR. Shown are mean values ± standard deviations obtained from three independent experiments, each performed in triplicate. All the differences (<50%) are considered to be insignificant. Download FIG S5, TIF file, 0.03 MB. Copyright © 2019 Lin et al. 2019 Lin et al. This content is distributed under the terms of the Creative Commons Attribution 4.0 International license.

10.1128/mBio.02401-18.6 FIG S6 Construction of TyL1 and TyL2 strains. The *murG* gene and its 5’ upstream DNA sequence (∼1 kb) in the R. sphaeroides wt or Δ*cls* genome are shown. In TyL1 and TyL2 genomes, a *gfp* gene is inserted between *murG* and its upstream sequence. The annealing sites and orientations of primers F (WSG) and R (HindIII-*murG1k*) are indicated. These primers were used to amplify chromosomal DNA of R. sphaeroides wt (lane 2), TyL1 (lane 3), R. sphaeroides Δ*cls* (lane 4), or TyL2 (lane 5) by PCR. The PCR products were analyzed by agarose gel electrophoresis. DNA standards (indicated in kilobases) are shown in lane 1. Download FIG S6, TIF file, 0.3 MB. Copyright © 2019 Lin et al. 2019 Lin et al. This content is distributed under the terms of the Creative Commons Attribution 4.0 International license.

Anionic phospholipids, such as CL and PGL, have been shown to accumulate at the polar regions of rod-shaped bacterial cells, where they may reduce the surface energy of curvature-mediated membrane strain (9, 28). We labeled R. sphaeroides membranes with the fluorescent probe 10-N-nonyl acridine orange (NAO), which has a preference for interacting with anionic phospholipids, and observed an enrichment of the fluorophore at the polar regions of both wt and Δ*cls* cells (Fig. 4C). As PGL is the precursor of CL, R. sphaeroides Δ*cls* cells contained a higher level of PGL than wt cells (11). Enrichment of MurG at the R. sphaeroides wt and Δ*cls* cell poles suggests that its localization may occur through interacting with CL and PGL. We purified recombinant R. sphaeroides MurG and quantified the interaction between MurG and membranes with different phospholipid compositions using *in vitro* liposome-pelleting assays. In these assays, 67% of the total MurG present interacted with liposomes containing 100 moles percent (mol%) PC and segregated to the pellet fraction. When 10 mol% CL or PGL was incorporated into PC-containing liposomes, the fraction of MurG interacting with membranes increased to 92% for both anionic phospholipids (Fig. 4D), suggesting equal preferences for MurG binding of CL and PGL. Other CL-binding peripheral membrane proteins show a similar relationship between these two lipids (29).

A hydrophobic patch in E. coli MurG was proposed to bind the cell membrane (30). Sequence alignment revealed that this membrane-binding interface is conserved in R. sphaeroides MurG (Fig. S7). The hydrophobic patch of amino acids in E. coli MurG is surrounded by several basic amino acid residues that may contribute to its preferential interaction with CL (30, 31). With the exception of R77 (corresponding to R79 in E. coli MurG), these residues are not conserved in R. sphaeroides MurG (Fig. S7). We purified R. sphaeroides MurG^R77E^ protein and found that the R77E mutation decreased the amount of MurG interacting with liposomes containing 100 mol% PC by 17% and abolished its preference for anionic phospholipids (Fig. 4D).

10.1128/mBio.02401-18.7 FIG S7 Multiple-sequence alignment of MurGs. Amino acid sequences of MurG from Staphylococcus aureus, E. coli, and R. sphaeroides were aligned using CLUSTAL O. Stars indicate conserved residues; colons indicate residues that are similar in size and hydropathy; periods indicate residues that are similar in size or hydropathy. Amino acids are highlighted: in red for residues involved in membrane binding, in magenta for residues involved in interaction with anionic phospholipids, and in yellow for residues involved in substrate binding. E. coli MurG contains a hydrophobic patch consisting of residues I74, L78, F81, W84, and W115, which is proposed to be the membrane association site. This membrane-binding patch is surrounded by several basic residues (K68, K71, R79, R85, R88, and K139). On the basis of the alignment, we propose that R. sphaeroides MurG also contains a hydrophobic patch (A72, L76, A79, V82, V113). Residues involved in murgocil binding are labeled with an “@” above the sequences. The tryptophan residues in R. sphaeroides MurG (W31, W147) are highlighted in blue. Download FIG S7, TIF file, 0.9 MB. Copyright © 2019 Lin et al. 2019 Lin et al. This content is distributed under the terms of the Creative Commons Attribution 4.0 International license.

CL and PGL are both important for the function of E. coli MurG; however, CL is more effective at stimulating the GTase activity of MurG (31). Although the MurG-membrane interaction is unlikely to be affected in R. sphaeroides Δ*cls* cells (due to the presence of PGL), the depletion of CL in the cell membrane should in principle decrease MurG activity and reduce the biosynthesis of lipid II available for cell elongation. To test this hypothesis, we treated R. sphaeroides cells with the MurG inhibitor murgocil (32). Regrettably, murgocil is specific to staphylococcal species and was inactive against R. sphaeroides due to amino acid differences between the two strains in the MurG-murgocil binding site (Fig. S7). Alternatively, we indirectly compared the amounts of lipid II produced by R. sphaeroides wt and Δ*cls* cells by quantifying the incorporation of HADA into the cell wall (Fig. 4E). Removing CL decreased the production of lipid II in R. sphaeroides by 25% (Fig. 4F). Treating R. sphaeroides cells with FOS and DCS reduced HADA incorporation by 21% and 24%, respectively (Fig. S8A). Complementation of CL synthase in R. sphaeroides Δ*cls* cells (which restores ∼50% of the concentration in wt cells and the rate of cell elongation by 20%; Fig. S1) restored lipid II production by 14% as measured in this assay (Fig. 4F), suggesting that CL is important for the enzymatic activity of MurG and cell elongation. Although murgocil is ineffective against Gram-negative bacteria, we performed a control experiment in which we treated Staphylococcus aureus cells with murgocil and found an 81% decrease in HADA incorporation (Fig. S8B), thereby correlating MurG activity to PG assembly.

10.1128/mBio.02401-18.8 FIG S8 Inhibition of lipid II production decreases HADA incorporation into the cell wall. (A) Quantification of HADA incorporation in R. sphaeroides wt cells, in wt cells treated with FOS, and in wt cells treated with DCS. Cells were grown in plain medium or in medium containing the indicated small-molecule inhibitors until early log phase (absorbance of 0.3, λ = 600 nm) and labeled with 0.5 mM HADA. We measured the fluorescence emission of HADA and normalized the fluorescent signals by O.D. Each data point (mean value ± standard deviation) was obtained from three independent experiments. The concentrations of inhibitors used were as follows: FOS, 250 μg/ml; DCS, 0.05 μg/ml. (B) Quantification of HADA incorporation in S. aureus cells treated with murgocil or the solvent control dimethyl sulfoxide (DMSO). Cells were grown until log phase (absorbance of 0.6, λ = 600 nm) and treated with DMSO or murgocil at a final concentration of 20 μg/ml (10× MIC) for 20 min. Cells were then labeled with 0.24 mM HADA for another 20 min. We washed the cells with PBS to remove excess dye and measured the fluorescence emission of HADA. Each data point (mean value ± standard deviation) was obtained from three independent experiments. Download FIG S8, TIF file, 0.03 MB. Copyright © 2019 Lin et al. 2019 Lin et al. This content is distributed under the terms of the Creative Commons Attribution 4.0 International license.

### Overexpression of MurG in R. sphaeroides Δ*cls* cells restores the rod shape of cells.

**FIG 5:**
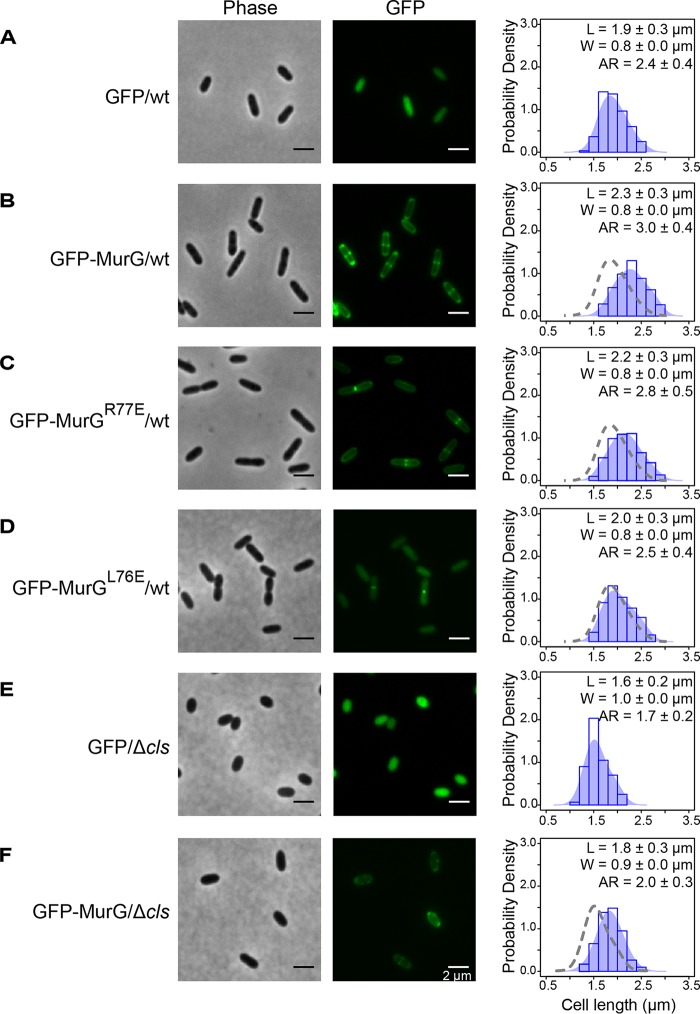
MurG activity correlates with the length of R. sphaeroides cells. The micrographs are representative of R. sphaeroides wt cells expressing GFP (A), wt cells expressing GFP-MurG (B), wt cells expressing GFP-MurG^R77E^ (C), wt cells expressing GFP-MurG^L76E^ (D), Δ*cls* cells expressing GFP (E), and Δ*cls* cells expressing GFP-MurG (F) from the pIND vector without IPTG induction. Cells were grown to log phase (absorbance of 0.6, λ = 600 nm) and imaged using phase-contrast bright-field and fluorescence microscopy. A probability density histogram of the cell length distribution of each strain is shown on the right panel. Each data point represents a mean value ± standard deviation of the cell length (L), width (W), and aspect ratio (AR) for 300 cells determined by ImageJ. The shaded blue area overlaying the histogram represents the Kernel density estimation (KDE) of the cell length distribution. We overlaid a gray dashed line outlining the KDE of the cell length distribution of R. sphaeroides wt cells expressing GFP with the histograms of wt cells expressing GFP-MurG, wt cells expressing GFP-MurG^R77E^, and wt cells expressing GFP-MurG^L76E^ for comparison. We also overlaid a gray dashed line outlining the KDE of the cell length distribution of R. sphaeroides Δ*cls* cells expressing GFP with the histogram of Δ*cls* cells expressing GFP-MurG for comparison.

To further explore the correlation between changes in MurG activity and cell length, we increased MurG activity in R. sphaeroides cells by overexpressing MurG (an N-terminal green fluorescent protein [GFP] fusion protein) from pIND. Leaky expression of GFP-MurG from the plasmid did not affect the subcellular localization of MurG and cell growth (as did 1 and 10 μM IPTG); consequently, we used leaky expression to increase the intracellular concentration of MurG. This approach increased R. sphaeroides cell length by 21% compared to control cells expressing cytoplasmic GFP. Expressing GFP-MurG^R77E^ (which should have reduced binding to the membrane) in wt R. sphaeroides cells abolished polar localization of GFP and increased the cell length by 16% compared to cells expressing GFP (Fig. 5A to C). These results correlate MurG activity with the length of R. sphaeroides cells.

We created R. sphaeroides MurG mutant L76E (corresponding to residue L78 in the proposed hydrophobic patch of E. coli MurG) and expressed GFP-MurG^L76E^ in R. sphaeroides cells by the leaky-expression approach. GFP-MurG^L76E^ was localized to the division plane, failed to bind the R. sphaeroides cytoplasmic membrane, and increased the length of R. sphaeroides cells by only 5% compared to cells expressing GFP, demonstrating the importance of MurG-membrane interaction for cell elongation (Fig. 5D).

To compensate for the decreased MurG activity in R. sphaeroides Δ*cls* cells, we also increased MurG expression in cells using a leaky promoter. MurG expression increased the length of R. sphaeroides Δ*cls* cells by 13% compared to Δ*cls* cells expressing GFP (Fig. 5E and F), suggesting that the morphological abnormality of the R. sphaeroides Δ*cls* mutant is a result of a decrease in MurG activity that reduces the supply of lipid II for cell elongation.

### CL deficiency increases the sensitivity of R. sphaeroides to antibiotics targeting PG synthesis.

**FIG 6:**
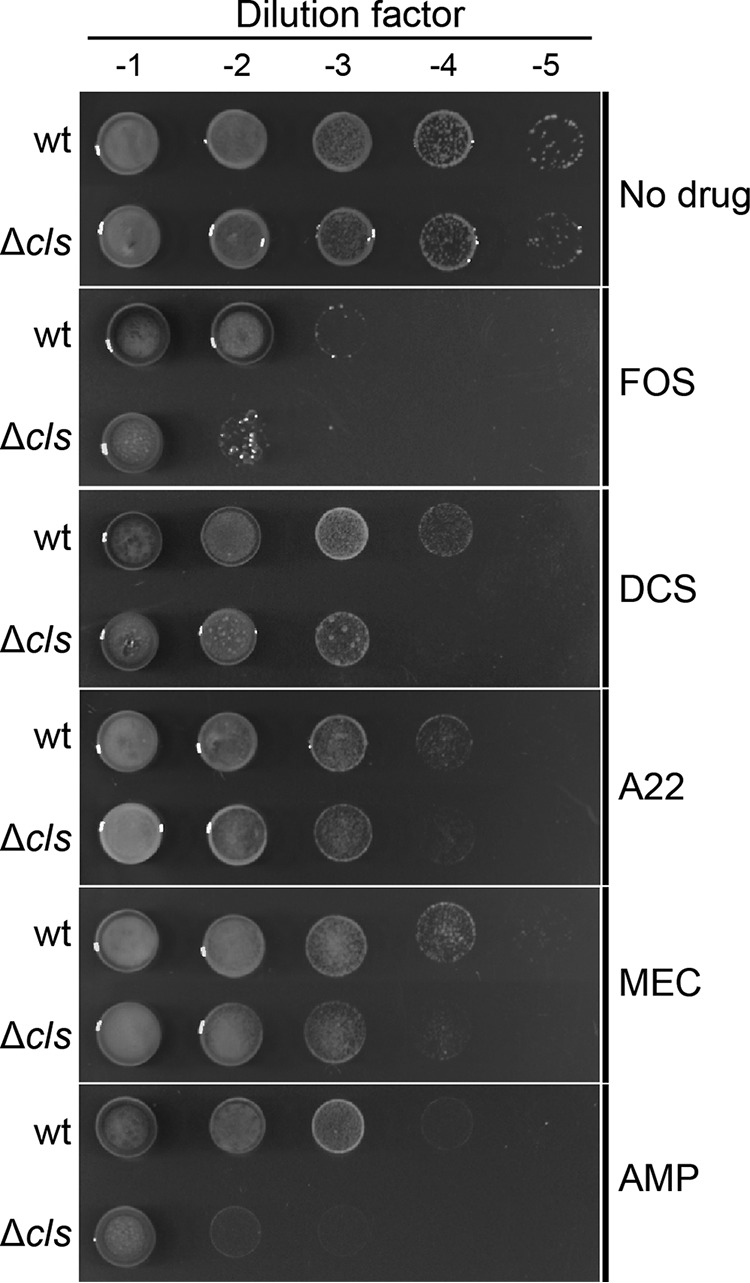
R. sphaeroides Δ*cls* mutant is susceptible to antibiotics targeting PG synthesis. Data represent results of spot-titer assays of R. sphaeroides wt and Δ*cls* cells. Cell cultures in stationary phase were standardized to an absorbance of 1.0 (λ = 600 nm). We serially diluted the standardized cultures and transferred 5-μl aliquots to the surface of plates containing Sistrom’s minimal medium with 1.5% agar and sub-MICs of the antibiotics indicated (FOS [125 μg/ml], DCS [0.05 μg/ml], A22 [5 μg/ml], MEC [0.1 μg/ml], and AMP [5 μg/ml]). Plates were incubated for 72 h at 30°C.

As CL alters the activity of MurG, which catalyzes the rate-limiting step in the biosynthesis of lipid II, we hypothesized that the R. sphaeroides Δ*cls* mutant may have increased susceptibility to antibiotics that inhibit lipid II biosynthesis and its assembly. To test this hypothesis, we performed spot-titer assays and found that R. sphaeroides Δ*cls* cells—compared to wt cells—had increased sensitivity to drugs interfering with lipid II biosynthesis, including FOS and DCS, and drugs interrupting PG assembly, including A22, MEC, and ampicillin (AMP; binds penicillin-binding proteins [PBPs] and inhibits their TPase activities) (Fig. 6). These results further support our hypothesis that a CL deficiency impedes the production of lipid II in R. sphaeroides.

## DISCUSSION

This study demonstrated that CL contributes to bacterial cell shape by altering cell wall synthesis. Our data led us to hypothesize that CL may regulate the activity of enzymes synthesizing lipid II during cell elongation. Of the nine lipid II synthases shown in Fig. 1, MurG catalyzes the rate-limiting step of lipid II biosynthesis and preferentially interacts with CL (versus PE and PGL) in E. coli (31). We demonstrated that the change in R. sphaeroides Δ*cls* cell shape arises from a decrease in MurG activity. A CL deficiency may also decrease the activities of other lipid II synthases in R. sphaeroides, such as the integral membrane protein MraY and the lipid II flippase MurJ. In addition to decreased cell length, R. sphaeroides cells that were Δ*cls* or treated with inhibitors FOS or DCS also had an increased cell width (Fig. 2B, E, and F), which suggests that inhibiting lipid II production may alter the orientation of MreB filaments and their relationship to cell diameter (33).

CL has four acyl tails and a relatively small head group that creates a large intrinsic curvature of ∼1.3 nm^−1^ (34). In rod-shaped bacteria, CL has a preference for localizing in the inner leaflet of the cell membrane and concentrates at regions of high membrane curvature (e.g., cell poles), where it reduces the local elastic strain on this liquid crystalline material (28); this phenomenon is particularly relevant to liquid crystals in the field of materials science (35). PGL has a significantly smaller curvature (∼0.11 nm^−1^) than CL and also concentrates at the polar regions of cells through unknown mechanisms, which may entail electrostatic interactions with proteins (9). The heterogeneous distribution of anionic phospholipids in membranes has been hypothesized to function as a mechanism for localizing membrane-associated proteins into different regions within bacterial cells (7). We found that MurG interacts with anionic phospholipids—equally preferring CL and PGL—and is enriched at the poles of R. sphaeroides cells, where PG is relatively inert. Polar enrichment of MurG has been observed in E. coli cells and has been hypothesized to provide a mechanism for the storage of excess protein (36).

Although we were unable to test it directly, our data support a model in which binding to CL influences the structure of MurG and stimulates its GTase activity. Within membranes that are energy transducing and have parallels to those found in R. sphaeroides, CL binds tightly to respiratory complexes in the electron transport chain, stabilizes protein complexes, and promotes ATP synthesis (37, 38). In E. coli, CL binds tightly to the SecYEG protein complex, stabilizes its dimeric form, and regulates protein translocation (39, 40). In Vibrio cholerae, CL stabilizes the oligomerization state of EpsE and stimulates its Eps system, which exports cholera toxin across the outer membrane (41). Purified, recombinant MurG and other GTases have been reported to be dimers or oligomers (42) that may be stabilized and activated by CL.

Although MurG also binds PGL and its subcellular localization in R. sphaeroides Δ*cls* cells is unchanged, PGL is unable to restore the observed reduction in MurG activity. A recent study demonstrated that E. coli RecA displays different tryptophan fluorescence emission spectra upon binding to CL- and PGL-containing liposomes and suggested that RecA adopts different conformations when interacting with different families or anionic phospholipids (43). To explore whether the structure of MurG may be altered upon membrane binding, we measured the tryptophan fluorescence of R. sphaeroides MurG (containing two tryptophan residues; see Fig. S7 in the supplemental material) and found difference in emission spectra in the presence of liposomes containing different anionic phospholipids (e.g., CL and PGL; Fig. S9). The structural mechanisms differentiating the effects of the two anionic phospholipids on MurG activity require further studies.

10.1128/mBio.02401-18.9 FIG S9 The tryptophan fluorescence emission spectrum of R. sphaeroides MurG changes when it binds to CL- and PGL-containing liposomes. MurG (7 μM) was incubated with 0.06 mM liposomes containing 10% CL or PGL (mol%) in Buffer T at room temperature for 15 min. The mixtures were excited with 280 nm UV light and scanned for fluorescence emission at 300 to 400 nm on a Tecan infinite M200 Pro microplate reader. We accounted for scattering of light generated by liposomes and corrected the fluorescence measurements by subtracting values of fluorescence of buffer containing liposomes only. Download FIG S9, TIF file, 0.04 MB. Copyright © 2019 Lin et al. 2019 Lin et al. This content is distributed under the terms of the Creative Commons Attribution 4.0 International license.

E. coli mutants lacking CL do not have an altered morphology because the MurG activity is increased in cells due to the upregulation of MurG expression (31). In contrast, the expression of MurG in R. sphaeroides Δ*cls* cells remained unchanged compared to R. sphaeroides wt cells (Fig. S10). Overexpression of MurG in R. sphaeroides wt cells increases the length of cells, correlating MurG activity to the growth of cells at the sidewalls. We overexpressed MurG in R. sphaeroides Δ*cls* cells to restore its activity and found that the cells partially recovered their rod-shaped morphology.

10.1128/mBio.02401-18.10 FIG S10 CL deficiency does not affect MurG protein expression in R. sphaeroides. TyL1 and TyL2 cell lysates that had the same amount of proteins were subjected to Western blot (WB) analysis using a monoclonal antibody against GFP. The amount of MurG in cells was determined by quantifying the optical densitometry signal using ImageJ. Data represent mean values ± standard deviations obtained from three independent experiments. Download FIG S10, TIF file, 0.02 MB. Copyright © 2019 Lin et al. 2019 Lin et al. This content is distributed under the terms of the Creative Commons Attribution 4.0 International license.

R. sphaeroides MurG mutant R77E interacts with the membrane, loses its preference for anionic phospholipids, and does not localize to cell poles. Expression of the MurG^R77E^ mutation increased the length of cells, suggesting that the mutation did not abolish MurG enzyme activity. However, the cells expressing MurG^R77E^ were shorter than the cells overexpressing wild-type MurG, which suggests that anionic phospholipids optimize the interaction of MurG with membranes and its activity. The molecular mechanisms underlying the preferential interaction between MurG and anionic phospholipids remain to be determined. Interestingly, the L76E MurG membrane-binding mutant was still able to localize at the division plane, suggesting that MurG interacts with the divisome using a motif that is different from the hydrophobic patch and does not interact with membranes during cell division. This observation may explain why a CL deficiency did not affect MurG activity at the septum and cause a defect in cell division in R. sphaeroides.

A growing body of evidence indicates that CL plays a fundamental role in a variety of bacterial processes, including ATP synthesis, DNA replication and repair, cell division, protein translocation, osmoadaptation, biofilm formation, and pathogenicity (7, 8, 29, 44–48). Our research provides the first example of the role of CL in determining bacterial cell shape. The PG layer of the cell wall is the canonical target for antibiotics; many of the antibiotics in this family have lost activities due to resistance. Although the R. sphaeroides Δ*cls* mutant has no significant defect in growth, it is more sensitive to antibiotics that target PG than the R. sphaeroides wt strain. It has been shown that a Pseudomonas putida Δ*cls* mutant is susceptible to several antibiotics, as its drug efflux system is impaired (49). These data suggest that in addition to the interesting biochemistry regulated by CL, its biosynthesis may be a potential target to explore for antibiotics or their adjuvants.

## MATERIALS AND METHODS

### Bacterial strains and growth conditions.

R. sphaeroides strains were grown aerobically in Sistrom’s succinate medium at 30°C with shaking at 200 rpm. When required, kanamycin (25 μg/ml), spectinomycin (5 μg/ml), or tetracycline (0.025 μg/ml) was added to the medium. E. coli strains were grown in LB broth at 37°C with shaking at 200 rpm. When required, kanamycin (25 μg/ml) or tetracycline (10 μg/ml) was added to the medium. The bacterial strains and plasmids used in this study are described in Table 2.

**TABLE 2:** Bacterial strains and plasmids used in this study

Strain or plasmid	Genotype or description	Source or reference
E. coli strains		
DH5α	*recA1 endA1 gyrA96 thi-1 hsdR17 supE44 relA1 deoR* Δ(*lacZYA-argF*)*U169 λ* (φ*80d lacZ*ΔM15)	Laboratory collection (CGSC#12384)
S17-1	*recA pro hsdR* RP4-2-Tc::Mu-Km::Tn7	Laboratory collection
BL21(DE3)	Host strain for recombinant protein expression	Laboratory collection
R. sphaeroides strains		
2.4.1	Wild type	ATCC 17023
Δ*cls*	2.4.1 containing a kanamycin resistance cassette in place of the genomic *cls*	11
TyL1	2.4.1 *murG*::*gfp-murG*	This study
TyL2	Δ*cls murG*::*gfp-murG*	This study

Plasmids		
pIND5	A derivative of pIND4 in which the NcoI site is replaced with an NdeI site; pIND4 is an IPTG-inducible expression vector for R. sphaeroides; kanamycin resistance	8
pIND5TetR	A variant of pIND5 with a tetracycline resistance cassette in place of the kanamycin resistance cassette	This study
*gfp-*pIND5	pIND5 containing *gfp*	This study
*gfp-murG*-pIND5	pIND5 containing *gfp-murG*	This study
*gfp-*pIND5TetR	pIND5TetR containing *gfp*	This study
*gfp-murG*-pIND5TetR	pIND5TetR containing *gfp-murG*	This study
*gfp-murG^R77E^*-pIND5	pIND5 containing *gfp-murG^R77E^*	This study
*gfp-murG^L76E^*-pIND5	pIND5 containing *gfp-murG^L76E^*	This study
pK18*mobsacB*	Suicide plasmid for allele exchange; kanamycin resistance	Laboratory collection
pK18*mobsacB*TetR	A variant of pK*18mobsacB* with a tetracycline resistance cassette in place of the kanamycin resistance cassette	This study
1k-*gfp-murG*-pK18 *mobsacB*TetR	pK18*mobsacB*TetR containing *murG* and its 5′ upstream DNA sequence, with a *gfp* inserted between the two	This study
pET28b(+)	A vector for expression of proteins with an N-terminal His tag/thrombin/T7 tag in the E. coli pET system	Novagen
*murG-*pET28b(+)	pET28b(+) containing *murG*	This study
*murG^R77E^-*pET28b(+)	pET28b(+) containing *murG^R77E^*	This study
*cls*-pIND5sp	pIND5sp containing *cls;* pIND5sp is a variant of pIND5 with a spectinomycin resistance cassette in place of the kanamycin resistance cassette	8

### Analysis of cell morphology.

An aliquot of cell cultures in log phase (absorbance of 0.6, λ = 600 nm) was transferred to the surface of a 2% (wt/vol) agarose pad prepared in phosphate-buffered saline (PBS) buffer (137 mM NaCl, 2.7 mM KCl, 10 mM Na_2_HPO_4_, 1.76 mM KH_2_PO_4_, pH 7.4), covered with a glass coverslip, and imaged with an inverted Nikon Eclipse Ti microscope equipped with a Photometrics CoolSNAP HQ2 charge-coupled-device (CCD) camera and a 120-W mercury arc lamp (X-cite Series 120; EXFO). Images were acquired with a 100× objective (Nikon Plan Apo 100×/1.40 oil Ph3 DM) and the Nikon Instruments Software (NIS)-Elements Advanced Research (AR) microscope imaging software program (version 4.000.07). Cell width and length were determined using ImageJ. To track the cell growth at single-cell level, cells were transferred to the surface of a 2% (wt/vol) agarose pad prepared in Sistrom’s succinate medium and imaged every 5 min for 6 h at 30°C.

### Plasmid constructions.

The primers used in this study are listed in Table 3. Cloning of PCR fragments into vectors were performed by In-Fusion cloning (Clontech) in accordance with the user manual unless otherwise noted. The pIND and pK18*mobsac*B constructs were transformed into E. coli S17-1 and subsequently mobilized via conjugation into the recipient R. sphaeroides wt or Δ*cls* strain as described previously (8).

**TABLE 3:** Primers used in this study

Primer name	Primer sequence
RF-TetR-F	TGCTTACATAAACAGTAATACAAGGGGTGTTATGAAACCCAACATACCCCTG
RF-TetR-R	GCCAGTGTTACAACCAATTAACCAATTCTGATCAGCGATCGGCTCGTTG
NdeI-*gfp*_p5	GGAGAAATTAACATATGGTGAGCAAGGGCGAGGAG
BglII-*gfp_*p5	GATGGTGATGAGATCTCTTGTACAGCTCGTCCAT
NdeI-*murG_*p5	GGAGAAATTAACATATGGGCCGGCCGCTCCTCCTG
BglII-*murG_*p5	GATGGTGATGAGATCTTGTCTCTTTCCTTGCCAG
*murG*-NdeI-*gfp*	AGCGGCCGGCCCATATGCTTGTACAGCTCGTCCAT
*murG*_R77E_S	CGCCGCCCGCGATCTCGAGGGGGACCAGC
*murG*_R77E_AS	GCTGGTCCCCCTCGAGATCGCGGGCGGCG
*murG*_L76E_S	GCCCGCGATGCGCTCGGGGACCAGCGC
*murG*_L76E_AS	GCGCTGGTCCCCGAGCGCATCGCGGGC
BamHI-*murG1k*	CGGTACCCGGGGATCCTCGACAAATGGTCGCTGA
HindIII-*murG1k*	GGCCAGTGCCAAGCTTTCATGTCTCTTTCCTTGC
*murG1k*+NdeI_S	GGGGCGGTGACCATATGGGCCGGCC
*murG1k*+NdeI_AS	GGCCGGCCCATATGGTCACCGCCCC
*murG1k-*NdeI*-gfp*	GGGGCGGTGACCATATGGTGAGCAAGGGCGAGGAG
NdeI-*murG*_p28	CGCGCGGCAGCCATATGGGCCGGCCGCTCCTCCTG
XhoI-*murG*_p28	GGTGGTGGTGCTCGAGTCATGTCTCTTTCCTTGC
WSG	AGATGGTCTATGGCTCCA

**(i) *gfp*-pIND5.** *gfp* was amplified by PCR using primers NdeI-*gfp*_p5 and BglII-*gfp*_p5 and cloned into pIND5 at the NdeI and BglII sites.

**(ii) *gfp-murG*-pIND5.** *murG* was amplified by PCR using primers NdeI-*murG*_p5 and BglII-*murG*_p5 from R. sphaeroides 2.4.1 genomic DNA and cloned into pIND5 at the NdeI and BglII sites to generate *murG*-pIND5. *gfp* was then amplified by PCR using primers NdeI-*gfp*_p5 and *murG*-NdeI-*gfp* and cloned into *murG*-pIND5 at the NdeI site.

**(iii) pIND5TetR.** The tetracycline resistance gene was amplified by PCR using primers RF-TetR-F and RF-TetR-R. The PCR products were used as megaprimers to replace the kanamycin resistance gene in pIND5 using the restriction-free method.

**(iv) *gfp*-pIND5TetR.** *gfp* was amplified by PCR using primers NdeI-*gfp*_p5 and BglII-*gfp*_p5 and cloned into pIND5TetR at the NdeI and BglII sites.

**(v) *gfp-murG*-pIND5TetR.** *murG* was amplified by PCR using primers NdeI-*murG*_p5 and BglII-*murG*_p5 from R. sphaeroides 2.4.1 genomic DNA and cloned into pIND5TetR at the NdeI and BglII sites to generate *murG*-pIND5TetR. *gfp* was then amplified by PCR using primers NdeI-*gfp*_p5 and *murG*-NdeI-*gfp* and cloned into *murG*-pIND5TetR at the NdeI site.

**(vi) *gfp*-*murG^R77E^*-pIND5.** *murG^R77E^* was created by using a Stratagene QuikChange XL site-directed mutagenesis kit in accordance with the manufacturer’s protocol. The template used was *gfp-murG*-pIND5, and the primers used were *murG*_R77E_S and *murG*_R77E_AS.

**(vii) *gfp-murG^L76E^*-pIND5.** *murG^L76E^* was created by using a Stratagene QuikChange XL site-directed mutagenesis kit in accordance with the manufacturer’s protocol. The template used was *gfp-murG*-pIND5, and the primers used were *murG*_L76E_S and *murG*_L76E_AS.

**(viii) 1k-*gfp*-*murG*-pK18*mobsacB*TetR.** *murG* and its 5′ upstream DNA sequence (∼1 kb) were amplified by PCR using primers BamHI-*murG1k* and HindIII-*murG1k* from R. sphaeroides 2.4.1 genomic DNA and cloned into pK18*mobsacB*TetR at the BamHI and HindIII sites to generate *1k*-*murG*-pK18*mobsacB*TetR. An NdeI site was inserted into *1k*-*murG*-pK18*mobsacB*TetR between *murG* and its 5′ upstream DNA sequence by the use of a Stratagene QuikChange XL site-directed mutagenesis kit and primers *murG1k*+NdeI_S and *murG1k*+NdeI_AS to create *1k*-NdeI-*murG*-pK18*mobsacB*TetR. *gfp* was then amplified by PCR using primers *murG1k*-NdeI-*gfp* and *murG-*NdeI*-gfp* and cloned into *1k*-NdeI-*murG*-pK18*mobsacB*TetR at the NdeI site.

**(ix) *murG*-pET28b(+).** *murG* was amplified by PCR using primers NdeI-*murG*_p28 and XhoI-*murG*_p28 and cloned into pET28b(+) at the NdeI and XhoI sites. The MurG expressed from the pET28b(+) vector contained an N-terminal His tag followed by a thrombin cleavage sequence.

**(x) *murG^R77E^*-pET28b(+).** *murG^R77E^* was created by using a Stratagene QuikChange XL site-directed mutagenesis kit in accordance with the manufacturer’s protocol. The template used was *murG*-pET28b(+), and the primers used were *murG*_L77E_S and *murG*_L77E_AS.

### qPCR.

Cells were grown to log phase (absorbance of 0.6, λ = 600 nm), and RNA was isolated as previously described (8). The RNA samples were then treated with RNase-free DNase (Qiagen, Valencia, CA) and further purified with an RNeasy CleanUp kit (Qiagen, Valencia, CA). cDNA synthesis was performed with a High Capacity RNA-to-cDNA kit (Applied Biosystems, Foster City, CA). Five nanograms of cDNA was used for each qPCR reaction. The Applied Biosystems 7500 real-time PCR system (Applied Biosystems, Foster City, CA) with SYBR green chemistry was used to monitor amplification and to quantify the amounts of PCR products. Relative quantitation of gene expression was calculated by the threshold cycle (ΔΔ*C_T_*) method; the level of *rpoZ* gene (encodes the Ω subunit of RNA polymerase) expression was used as an internal control. The qPCR primers used in this study are listed in Table 4.

**TABLE 4:** qPCR primers used in this study

Gene	Primer type	Primer sequence
*rpoZ*	Forward	TGACAAGAACCCTGTCGTG
	Reverse	GCAGCTTCTCTTCGGACAT

*mreB*	Forward	CTTCTTGCCGTCCTTCACA
	Reverse	CGCGAACACGCTGATCTA

*mreC*	Forward	CTTCCTTCCAGGCCTTCATC
	Reverse	TCGAGAACTTCCAGTCCTACA

*mreD*	Forward	CACATGCGTCTCCCGAAG
	Reverse	CCATCCTCTGCTATCCTCTGAT

*pbp2*	Forward	GATGACGACGCGGTAATTCT
	Reverse	ATCAACATCCGGCTGATCC

*rodA*	Forward	GTTCACCGCGAGATAGAAGAAG
	Reverse	CTCGCTTCTGGTGCTCTATG

*pbp1A*	Forward	CGTCATGAAGCTCTGGAACA
	Reverse	CGTCTGGTTCATCGGCTATAC

*murA*	Forward	CTTCCGCACGATGTCGTAAT
	Reverse	CACGATGACGCAGCTTCT

*murB*	Forward	GCATTCCAACTTCCTCATCAAC
	Reverse	CCGCATGATTTCCCACTCTA

*murC*	Forward	GCAAGACCACGACCACTAC
	Reverse	GAGCCATAGGCATGGATCAC

*murD*	Forward	CAATGACATCGGCCTCTTCT
	Reverse	CGACAGGATGTGATGGATGAG

*murE*	Forward	CGCATCGTCGTCGTCTTC
	Reverse	GATTGTCGTCGGTGACATAGAG

*murF*	Forward	AAGACCTCGACCAAGGAGA
	Reverse	CCCAGTGGTTGTTGTAGGAG

*ddlA*	Forward	CTCTTCGGTAGGCGTCTATATC
	Reverse	GACATAGGTCTCGACCATCAG

*mraY*	Forward	GCTCGACAATCCTTACGTCTG
	Reverse	CCCTTGGTGTTCTGCTTCTT

*murG*	Forward	ACCTTCTTCACCGACATTCC
	Reverse	CGGCCGATGATCGAGATG

*murJ*	Forward	CTCATGACCTTCCAGCTTCTT
	Reverse	ATGTTCCGCCGCTTCTT

### HADA labeling and quantification.

Cells were grown to early log phase (absorbance of 0.3, λ = 600 nm) and labeled with 0.5 mM HADA for ∼1/8 of the doubling time (20 min) at room temperature, followed by three washes with PBS to remove excess dye. We imaged the labeled cells by microscopy using a DAPI (4′,6-diamidino-2-phenylindole) filter. The fluorescence of HADA incorporated into PG was measured with an excitation wavelength of 405 nm and an emission wavelength of 450 nm using a Tecan infinite M200 Pro microplate reader (Tecan, San Jose, CA).

### PG isolation and UPLC-MS analysis.

Cells were grown in plain medium or medium containing the indicated small-molecule inhibitors (A22 [10 μg/ml], MEC [0.5 μg/ml], FOS [250 μg/ml], and DCS [0.05 μg/ml]) to early log phase (absorbance of 0.3, λ = 600 nm) and pelleted by centrifugation at 4,000 × *g* for 10 min at room temperature. Cells were then resuspended in 1 ml 0.25% SDS–0.1 M Tris/HCl (pH 6.8) and boiled for 40 min. The cell suspension was centrifuged at 10,000 × *g* for 5 min at room temperature, and the pellet was washed with double-distilled water (ddH_2_O) to remove SDS. The pellet was then resuspended in 1 ml ddH_2_O and put in a water bath sonicator for 30 min at room temperature. Nuclease solution (15 μg/ml DNase, 60 μg/ml RNase, 0.1 M Tris/HCl, pH 6.8) (500 μl) was added into the sample, which was then incubated for 60 min at 37°C in a shaker. The sample was augmented with 500 μl trypsin solution (50 μg/ml trypsin–ddH_2_O), incubated for another 60 min at 37°C in a shaker, and then boiled for 15 min to inactivate the enzymes. The suspension was centrifuged at 10,000 × *g* for 5 min at room temperature, and the pellet was washed with ddH_2_O. The pellet was resuspended in digestion buffer (12.5 mM sodium dihydrogen phosphate, pH 5.5) to reach an level of absorbance of 3.0 (λ = 600 nm), and a 1/10 volume of mutanolysin solution (5.000 U/ml mutanolysin–ddH_2_O) was added. The sample (∼175 μl) was incubated for 16 h at 37°C in a shaker and then boiled for 15 min to inactivate the enzyme. The suspension was centrifuged at 10,000 × *g* for 5 min at room temperature, and the PG was in the supernatant. A 50-μl volume of reduction solution (20 mg/ml sodium borohydrate–0.5 M borax–ddH_2_O, pH 9.0) was added into the PG solution, which was then incubated for 30 min at room temperature. The sample was adjusted to pH 2.0 to 3.0 with phosphoric acid solution (50%) and analyzed by UPLC-MS.

For UPLC-MS, we injected 7.5 μl of purified muropeptides into a Cortecs C_18_ column (Waters) (2.1 by 100 mm) packed with 1.6-μm-diameter particles and equipped with a Cortecs C_18_ guard column (Waters). The column temperature was maintained at 52°C using an Acquity standard flow UPLC system equipped with an inline photodiode array (Waters). For muropeptide separation by UPLC, we used solvent A (Optima LC-MS-grade water–0.05% trifluoroacetic acid) and solvent B (30% [vol/vol] Optima LC-MS-grade methanol–Optima LC-MS-grade water–0.05% trifluoroacetic acid) (Fisher Scientific). Muropeptides were eluted from the column with a gradient of increasing solvent B (1 min hold at 1% B to 99% B at 60 min to hold at 99% B 5 min to 1% B at 65.5 min to 4.5 min hold at 1% B) at a flow rate of 0.2 ml/min. We analyzed the eluent from the column using a Bruker MaXis Ultra-High Resolution time-of-flight 4G mass spectrometer (Bruker Daltonik) with an MS method. The capillary voltage was set to 4,100 V, the nebulizer pressure was 2.0 bar, and the drying gas was set to 6.0 liters/m at 220°C. Muropeptides were detected at λ = 205 nm and via MS.

### Creation of TyL1 and TyL2.

1k-*gfp*-*murG*-pK18*mobsacB*TetR was introduced into R. sphaeroides wt or Δ*cls* cells by conjugation. The integration of plasmid into the R. sphaeroides genome through homologous recombination was selected by plating cells on plates containing Sistrom’s minimal medium with 1.5% agar and tetracycline. A single colony was picked into plain Sistrom’s minimal medium and grown for 2 days. The excision of the plasmid from the genome through a second homologous recombination step was selected by plating cells on plates containing Sistrom’s minimal medium with 1.5% agar and 10% sucrose. The insertion of *gfp* between *murG* and its upstream sequences was confirmed by PCR using primers WSG and HindIII-*murG1k*.

### Cell fractionation.

Cells were grown to early log phase (absorbance of 0.3, λ = 600 nm), pelleted by centrifugation at 5,000 × *g* for 10 min at 4°C, and then resuspended in Buffer T (20 mM Tris-Cl, pH 8.0). Cells were passed through a Constant Systems cell disruptor (Constant Systems Ltd.) at 10,000 lb/in^2^, and the resulting cell lysate was clarified by centrifugation at 5,000 × *g* for 10 min at 4°C. The clear lysate was quantified, and equal amounts of wt and Δ*cls* lysates were brought to the same volume using Buffer T, followed by centrifugation at 45,000 × *g* for 30 min at 4°C, and were then divided into fractions of supernatant and pellet (dissolved in Buffer T). The same amounts of proteins in the fractions of supernatant (enriched in cytosolic proteins) and pellet (enriched in membrane proteins) were subjected to Western blot analysis using anti-GFP antibodies.

### NAO staining.

Cells were grown to early log phase (absorbance of 0.3, λ = 600 nm), and NAO was added at a final concentration of 10 μM into the cultures, which were grown for another 4 h. The cells were then imaged under a fluorescence microscope using 480-nm-excitation and 685-nm-emission filters.

### MurG purification.

E. coli BL21(DE3) cells transformed with pET28b containing R. sphaeroides *murG* were grown to an absorbance of 0.6 (λ = 600 nm) and induced with 1 mM IPTG for 3 h at 37°C. Cells were pelleted by centrifugation at 4,000 × *g* for 10 min at 4°C and then resuspended in Buffer T containing 5 mM imidazole and 0.1 mM phenylmethylsulfonyl fluoride (PMSF). Cells were passed through a Constant Systems cell disruptor (Constant Systems Ltd.) at 20,000 lb/in^2^, and the resulting cell lysate was mixed with Triton X-100 at a final concentration of 3% for 1 h at 4°C with rotation. The supernatant and insoluble fractions were separated by centrifugation at 25,000 × *g* for 40 min at 4°C. The supernatant was added to a nickel-nitrilotriacetic acid (Ni-NTA) agarose (Qiagen) column equilibrated with Buffer T. The column was washed stepwise with Buffer T, 25 imidazole–Buffer T, and then 50 mM imidazole–Buffer T, and the MurG protein was eluted with 100 mM imidazole–Buffer T. The protein was concentrated into Buffer T (without imidazole) using a 10-kDa-cutoff spin column (Millipore). To remove the His tag from the recombinant protein, the protein concentrate was subjected to thrombin (Millipore) cleavage with a dilution factor for thrombin of 1:200 for 16 h at 22°C. The reaction mixture was mixed with 25 μl of streptavidin agarose beads at 22°C for 30 min with rotation, followed by centrifugation in a spin filter (Millipore). The filtrate, free of biotinylated thrombin, was concentrated into Buffer T using a 10-kDa-cutoff spin column. To remove undigested protein, 25 μl of Ni-NTA agarose was added to the resulting protein concentrate and the sample was incubated at 22°C for 30 min with rotation. The Ni-NTA agarose was removed using a spin filter. The concentration of MurG with a molar extinction coefficient of 21,430 cm^−1^ M^−1^ was determined by measuring the absorbance at λ = 280 nm.

### Liposome preparation.

The following phospholipids used in this study were purchased from Avanti Polar Lipids: 1,2-di-(9Z-octadecenoyl)-*sn*-glycero-d-phosphocoline (PC), 1,2-di-(9Z-octadecenoyl)-*sn*-glycero-d-phospho-(1′-rac-glycerol) (PG), and 1,1′,2,2′-tetra-(9Z-octadecenoyl) cardiolipin (CL). To prepare liposomes of uniform size, lipid mixtures were dissolved in chloroform, dried in a glass vial, and rehydrated in Buffer T. The resulting liposome solutions were then frozen in liquid nitrogen and thawed in a 37°C water bath 5 times, followed by extrusion (Avanti Polar Lipid Mini Extruder) 15 times through a membrane filter (Whatman) with a pore size of 1 μm.

### Liposome-pelleting assay.

To block nonspecific binding, phospholipids at 5 mM were preincubated with 0.6 μM of bovine serum albumin (BSA)–Buffer T for 10 min. MurG in Buffer T was then added into the solution, resulting in a final MurG concentration of 6 μM, a phospholipid concentration of 4 mM, and a BSA concentration of 0.5 μM. The reaction mixture was incubated for 30 min, centrifuged at 16,300 × *g* for 30 min, and then divided into fractions of supernatant and pellet (dissolved in Buffer T). All the procedures were performed at room temperature. Proteins in the supernatant and pellet fractions were subjected to SDS-PAGE and visualized by Coomassie blue staining.
