# Supplementary figures and images for: Cardiolipin Alters Rhodobacter sphaeroides Cell Shape by Affecting Peptidoglycan Precursor Biosynthesis
Source: mBio. 2019 Feb 19;10(1):e02401-18. doi: 10.1128/mBio.02401-18 (PMC6381277; doi:10.1128/mBio.02401-18)

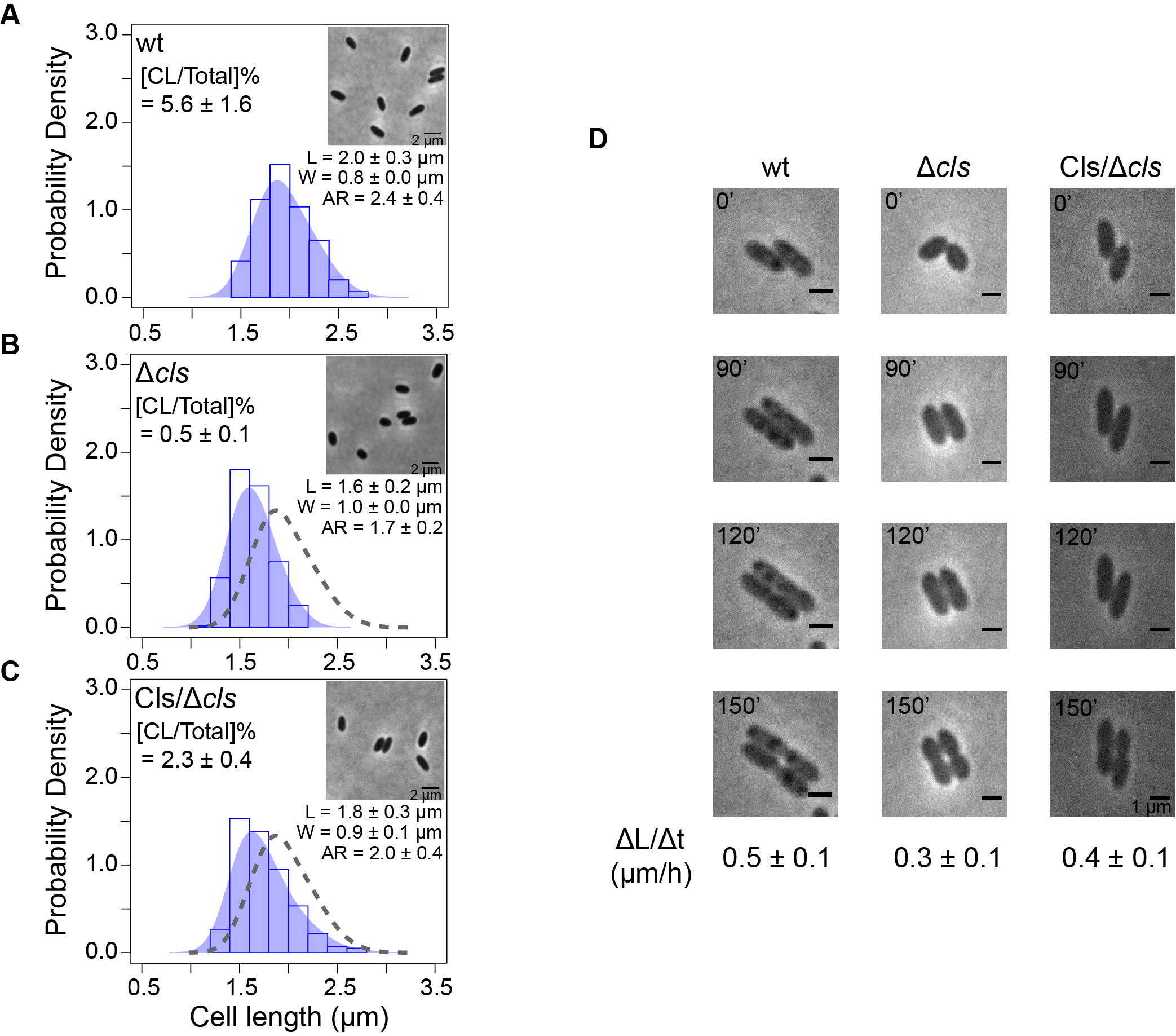

Supplement: FIG S1 [file mBio.02401-18-sf001.tif]

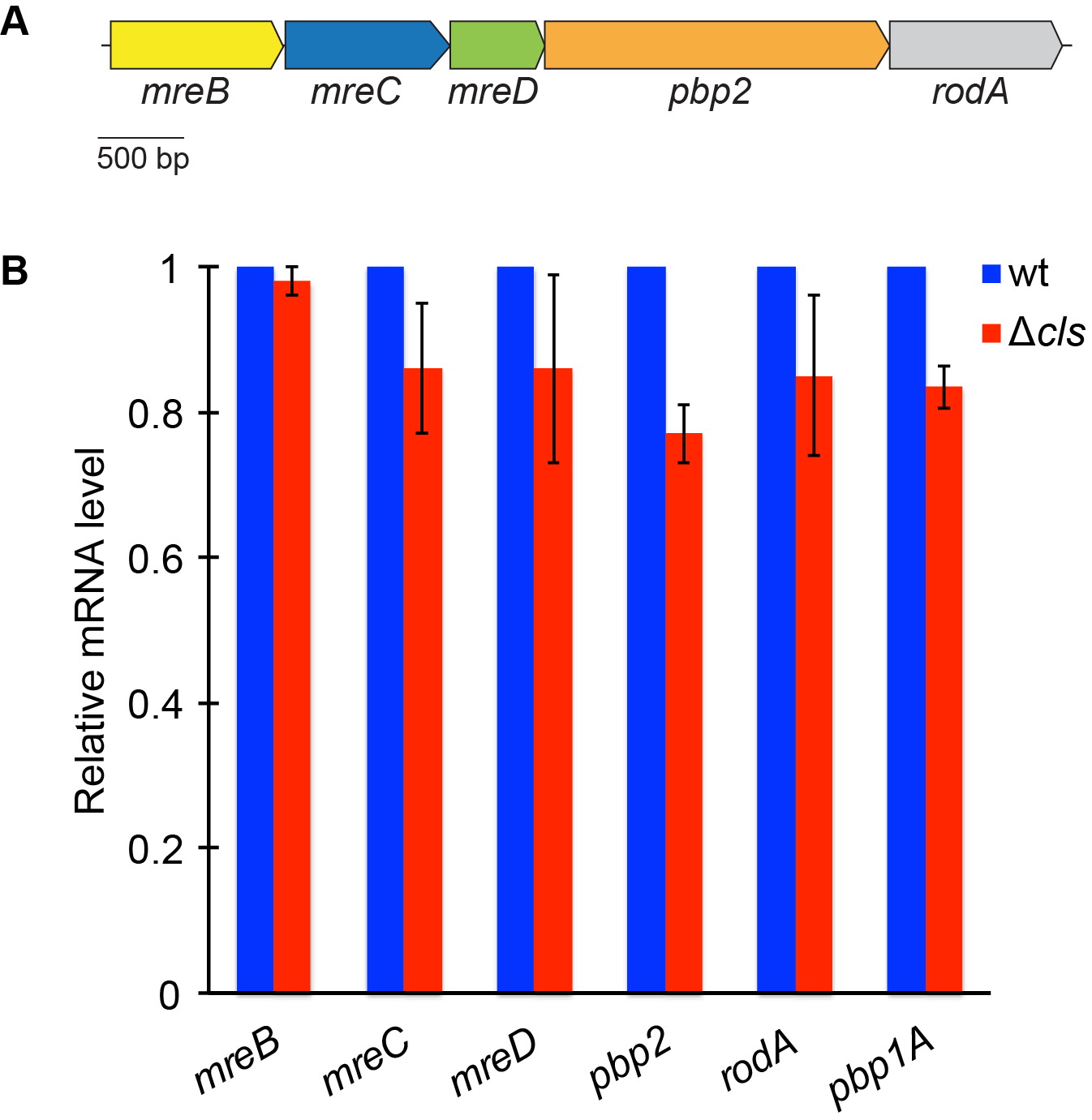

Supplement: FIG S2 [file mBio.02401-18-sf002.tif]

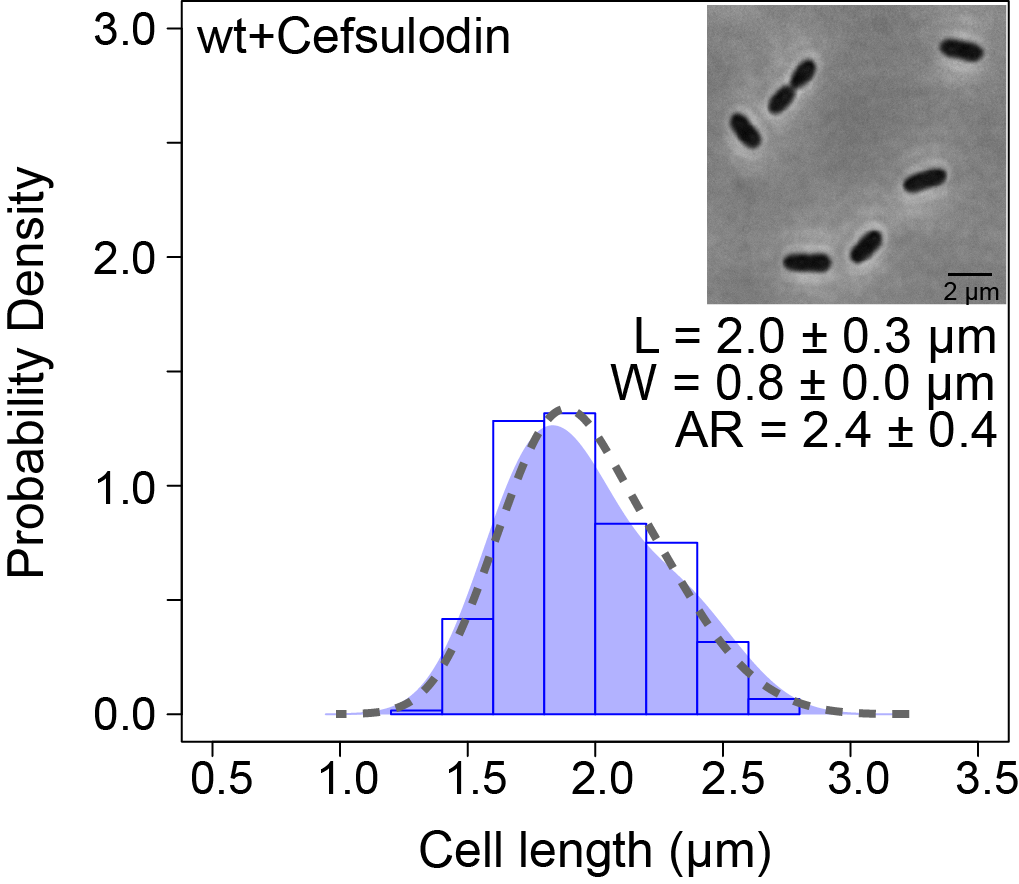

Supplement: FIG S3 [file mBio.02401-18-sf003.tif]

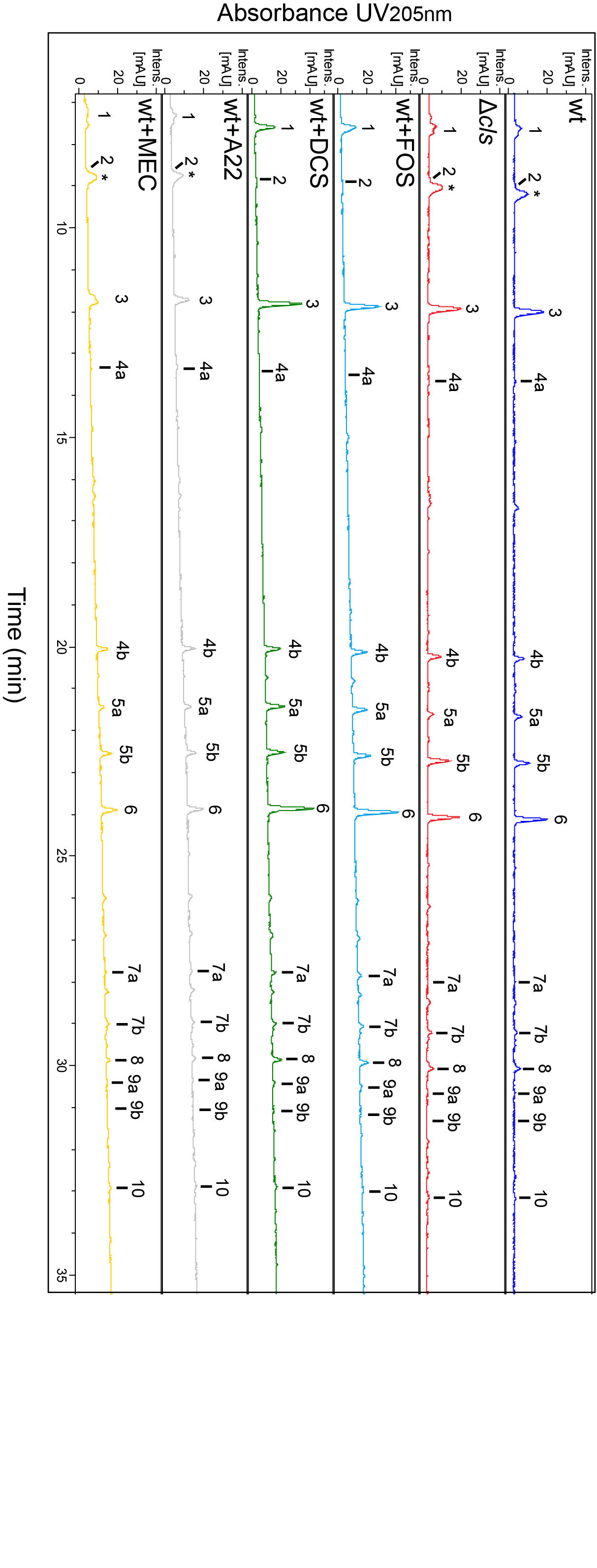

Supplement: FIG S4 [file mBio.02401-18-sf004.tif]

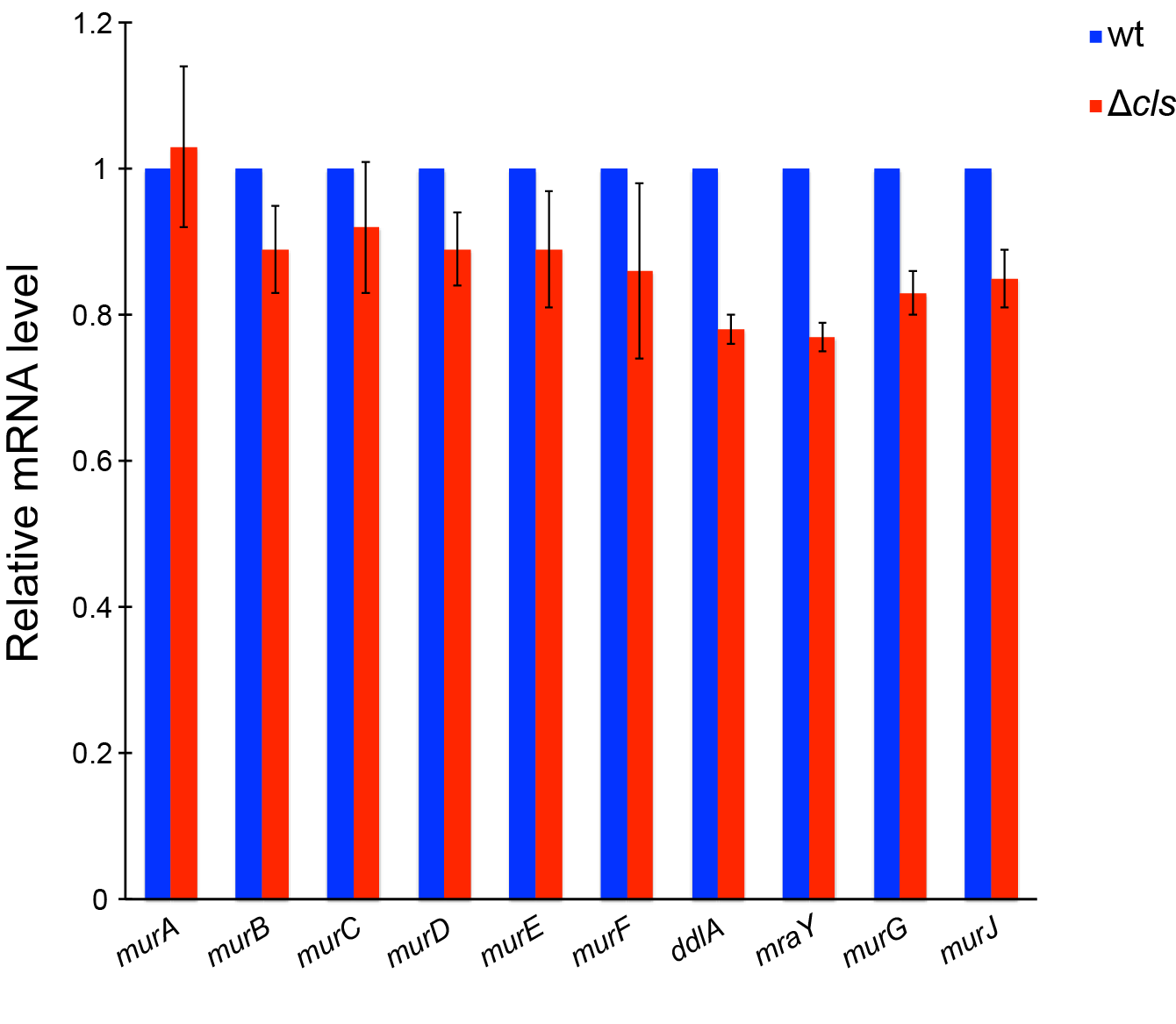

Supplement: FIG S5 [file mBio.02401-18-sf005.tif]

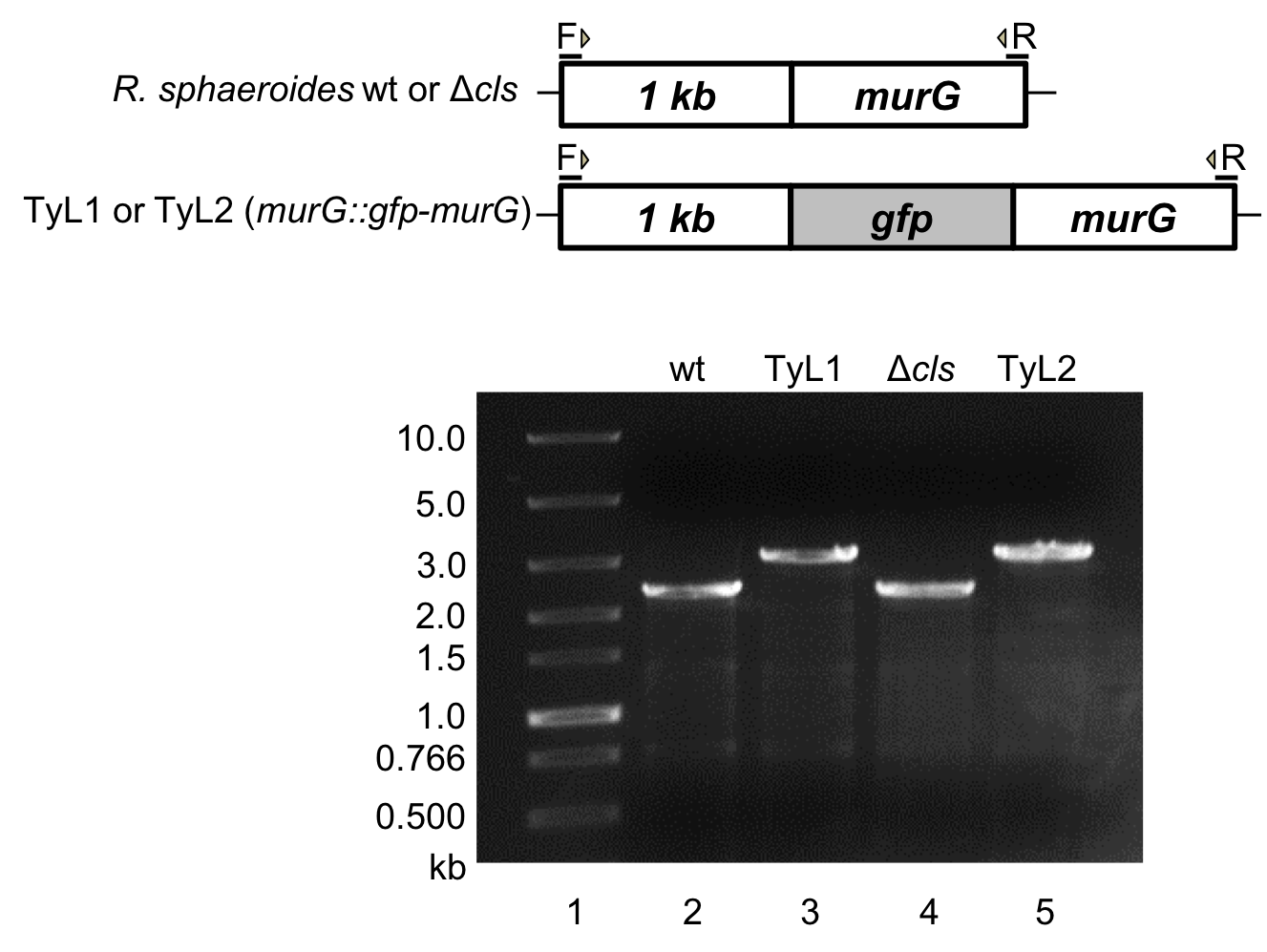

Supplement: FIG S6 [file mBio.02401-18-sf006.tif]

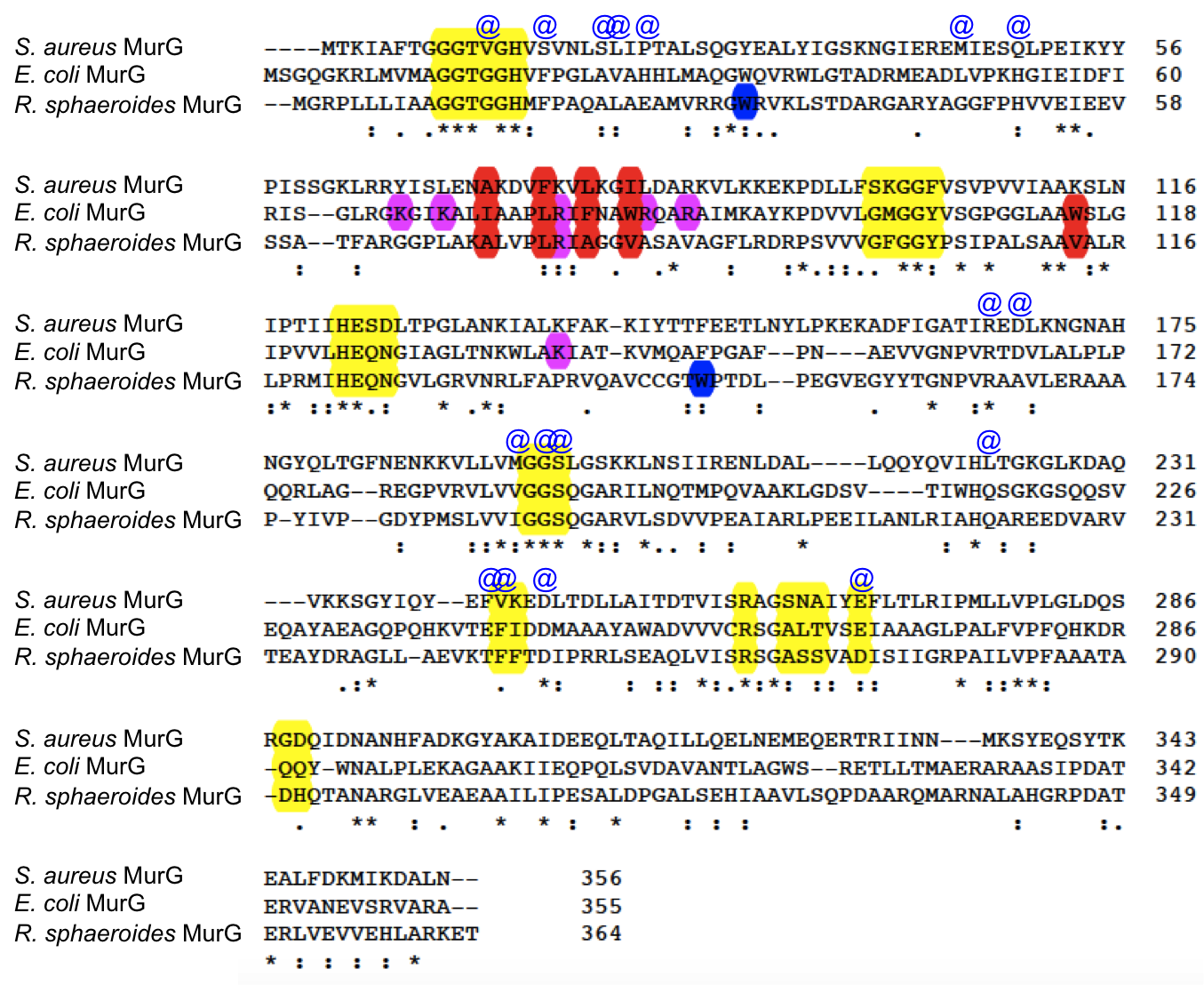

Supplement: FIG S7 [file mBio.02401-18-sf007.tif]

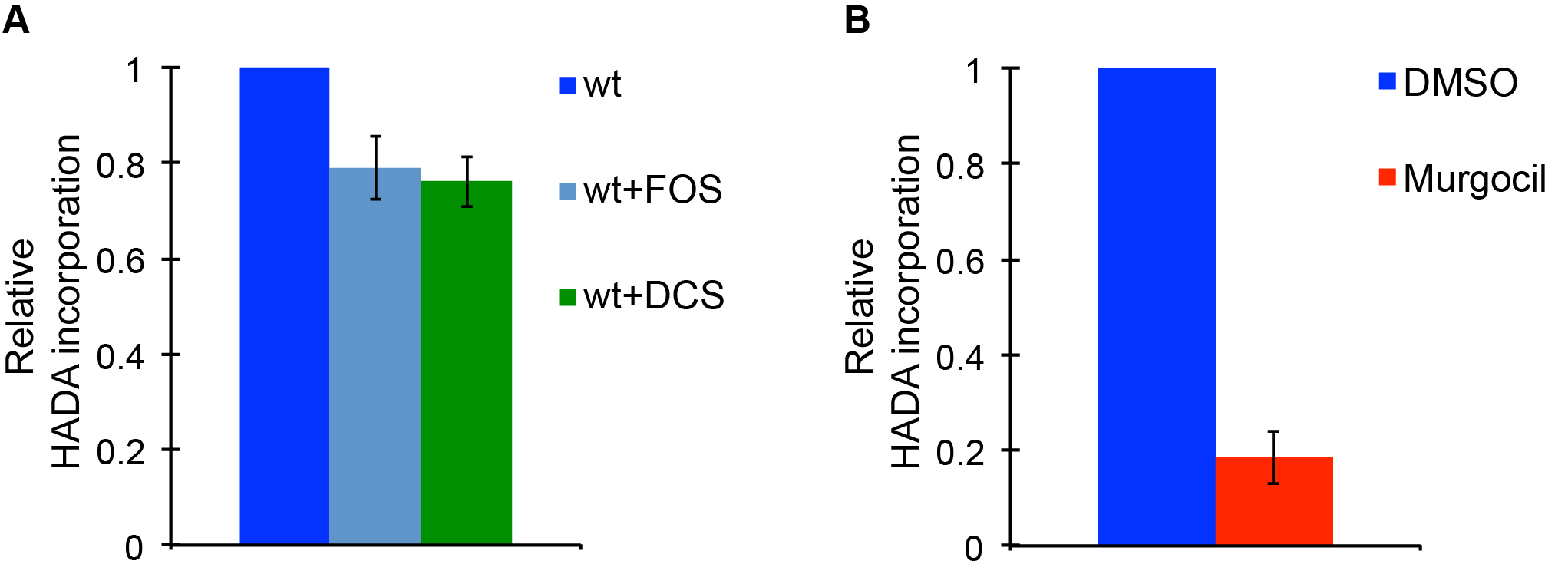

Supplement: FIG S8 [file mBio.02401-18-sf008.tif]

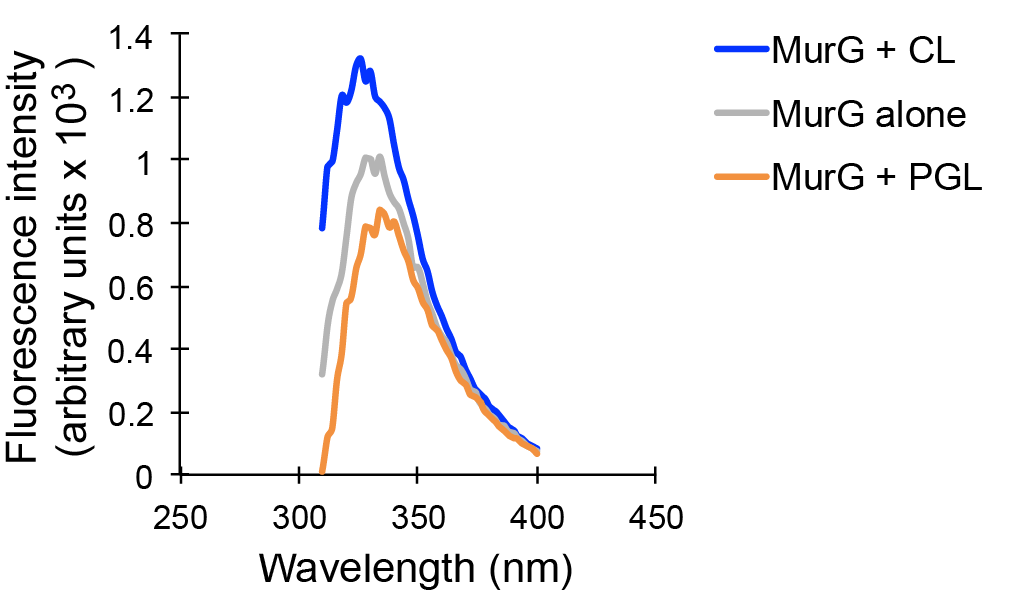

Supplement: FIG S9 [file mBio.02401-18-sf009.tif]

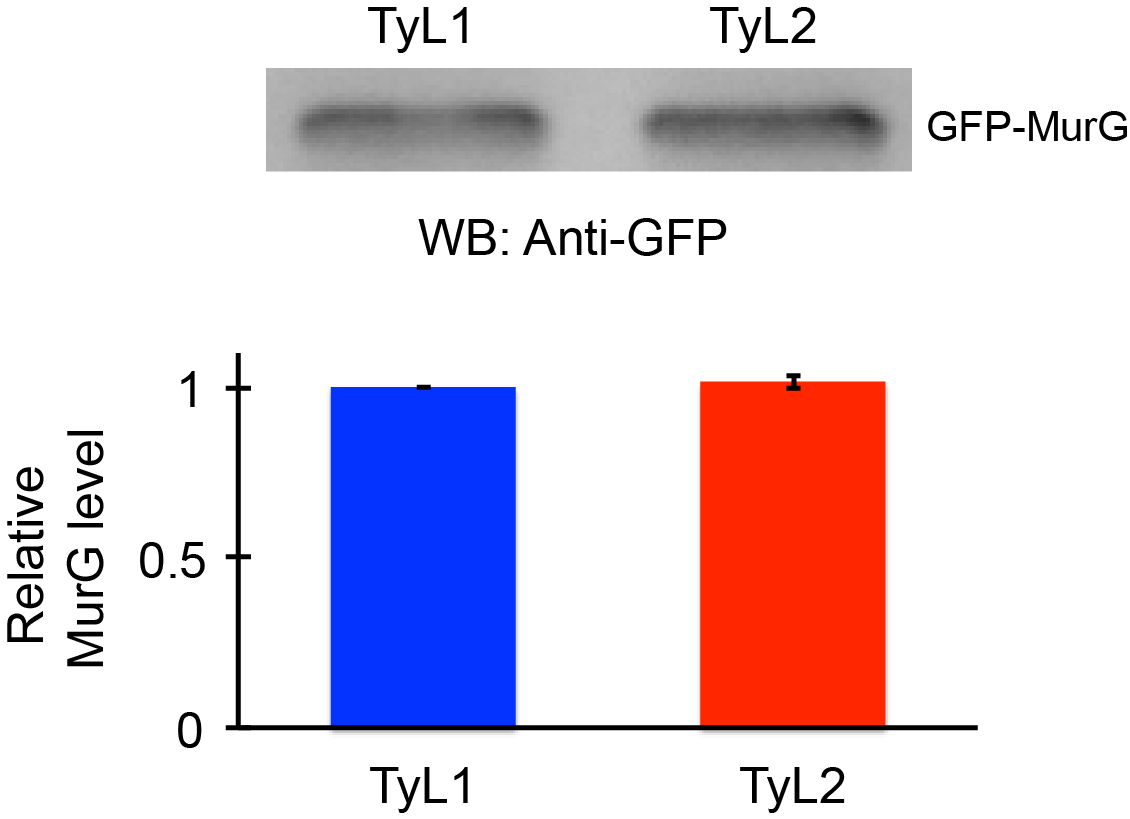

Supplement: FIG S10 [file mBio.02401-18-sf010.tif]
